# Rab35-regulated lipid turnover by myotubularins represses mTORC1 activity and controls myelin growth

**DOI:** 10.1038/s41467-020-16696-6

**Published:** 2020-06-05

**Authors:** Linda Sawade, Federica Grandi, Marianna Mignanelli, Genaro Patiño-López, Kerstin Klinkert, Francina Langa-Vives, Roberta Di Guardo, Arnaud Echard, Alessandra Bolino, Volker Haucke

**Affiliations:** 1 Leibniz-Forschungsinstitut für Molekulare Pharmakologie (FMP), Robert-Rössle-Strasse 10, 13125 Berlin, Germany; 2 Institute of Experimental Neurology (InSpe), Division of Neuroscience, IRCCS Ospedale San Raffaele, Via Olgettina 60, 20132 Milan, Italy; 3 San Raffaele Vita-Salute University, Via Olgettina 60, 20132 Milan, Italy; 4 Laboratorio de Investigación en Inmunología y Proteómica, Hospital Infantil de México, Federico Gómez. C.P, 06720 Ciudad de México, México; 5 Membrane Traffic and Cell Division Lab, Institut Pasteur, UMR3691, CNRS, 25–28 rue du Dr Roux, F-75015 Paris, France; 6 Sorbonne Université, Collège doctoral, F-75005 Paris, France; 7 Centre d’Ingénierie Génétique Murine, Institut Pasteur, 25–28 rue du Dr Roux, F-75015 Paris, France; 8 Freie Universität Berlin, Faculty of Biology, Chemistry and Pharmacy, 14195 Berlin, Germany; 9 Charité – Universitätsmedizin Berlin, corporate member of Freie Universität Berlin and Humboldt- Universität zu Berlin, NeuroCure Cluster of Excellence, 10117 Berlin, Germany

**Keywords:** Lipid signalling, Nutrient signalling, Small GTPases, Myelin biology and repair

## Abstract

Inherited peripheral neuropathies (IPNs) represent a broad group of disorders including Charcot-Marie-Tooth (CMT) neuropathies characterized by defects primarily arising in myelin, axons, or both. The molecular mechanisms by which mutations in nearly 100 identified IPN/CMT genes lead to neuropathies are poorly understood. Here we show that the Ras-related GTPase Rab35 controls myelin growth via complex formation with the myotubularin-related phosphatidylinositol (PI) 3-phosphatases MTMR13 and MTMR2, encoded by genes responsible for CMT-types 4B2 and B1 in humans, and found that it downregulates lipid-mediated mTORC1 activation, a pathway known to crucially regulate myelin biogenesis. Targeted disruption of Rab35 leads to hyperactivation of mTORC1 signaling caused by elevated levels of PI 3-phosphates and to focal hypermyelination in vivo. Pharmacological inhibition of phosphatidylinositol 3,5-bisphosphate synthesis or mTORC1 signaling ameliorates this phenotype. These findings reveal a crucial role for Rab35-regulated lipid turnover by myotubularins to repress mTORC1 activity and to control myelin growth.

## Abstract (web summary)

Charcot-Marie-Tooth (CMT) is an inherited peripheral neuropathy. Here, the authors show that Rab35 forms a complex with genes implicated in CMT, MTMR13 and MTMR2, which regulates myelin growth by controlling mTORC1 signaling through lipid turnover.

## Introduction

Inherited peripheral neuropathies (IPNs) represent a broad group of genetically and clinically heterogeneous disorders, including Charcot-Marie-Tooth (CMT) neuropathies, one of the most common inherited neurological disorders^1^. Within the past decades almost 100 IPN/CMT disease genes have been identified, yet, the molecular mechanisms underlying most of the IPN/CMT neuropathies are incompletely understood. Among CMTs, CMT4B is characterized pathologically by the presence of myelin outfoldings, a form of focal hypermyelination with redundant loops of myelin^2^. CMT4B includes three distinct subtypes. CMT4B1 and CMT4B2 are associated with mutations in the *MTMR2* and *MTMR13* (myotubularin-related protein 2 and 13, the latter also named SET binding factor 2, *SBF2*) genes, respectively, and are characterized by a demyelinating neuropathy with early onset^3,4^. CMT4B3 has been more recently associated with mutations in the *MTMR5/SBF1* gene but is characterized by different phenotypes with either a pure demyelinating neuropathy or an axonal polyneuropathy complicated by central nervous system involvement^2^. The tissue specificity of CMT4B disease phenotypes suggests that MTMR2, MTMR5, and MTMR13 have cell-type specific functions. MTMR2 is a ubiquitously expressed phosphatidylinositol 3-phosphatase of the myotubularin-related protein family that dephosphorylates both phosphatidylinositol 3-phosphate [PI(3)P] and phosphatidylinositol 3,5-bisphosphate [PI(3,5)P_2_] phospholipids, which are mainly enriched in the endolysosomal system^5,6^. Consistently, we found that PI(3,5)P_2_ levels are increased in primary cells from *Mtmr2* KO mutant mice, which recapitulate CMT4B1 in humans, suggesting that this lipid is an important substrate of MTMR2 in Schwann cells in vivo^7^. On the contrary, MTMR5 and MTMR13 are catalytically inactive proteins and associate with MTMR2 to potentiate phosphatase activity and to regulate its subcellular localization^8,9^. The localization of these MTMRs, however, remains to be clearly defined. How elevated levels of phosphatidylinositol (PI) 3-phosphates under conditions of loss-of-function of MTMR2 and/or MTMR5/MTMR13 may perturb myelination in the peripheral nervous system is largely unknown.

Recent data from non-myelin forming cell types suggest that PI(3)P and PI(3,5)P_2_ locally facilitate nutrient signaling by mTORC1 at late endosomes and lysosomes^10–13^. Elevated signaling via the AKT-mTORC1 axis, e.g. upon constitutive AKT1 activation or conditional genetic disruption of PTEN in Schwann cells causes focal hypermyelination consisting of redundant loops of myelin and tomacula^14,15^, while hyperactive mTORC1 during early stages of development delays the onset of myelination^16^. Loss of mTORC1 activity has been shown to hamper myelination^17,18^. These data suggest that mTORC1 signaling plays a dual role in controlling myelination in the peripheral nervous system^19^ that may conceivably be modulated by PI 3-phosphates that serve as substrates for MTMRs.

The small GTPase Rab35, a central regulator of endosomal function^20,21^ has been implicated in a variety of cell physiological pathways that range from the regulation of endosomal trafficking^20–22^ including secretion of exosomes^23^, actin dynamics^21^ and apico-basal polarity^24^ to cytokinesis^25,26^ and the modulation of cell signaling^27^, and migration^24,28,29^. These various roles have been linked to the ability of Rab35 to bind and recruit effector proteins such as the PI 5-phosphatase OCRL^30,31^, the Arf6 GTPase activating protein ACAP2^32,33^, the oxidoreductase MICAL1^34^ and the endosomal protein MICAL-L1^35^. Given the multitude of effector proteins for other endosomal Rabs such as Rab5 it is likely that additional Rab35 effector proteins exist. Rab35 activation is triggered by GEFs including endocytic or endosomal DENN domain-containing proteins^20,30,36^ and, possibly, the late endosomal/lysosomal mTORC1 regulator folliculin, which contains a DENN-like module^37,38^.

Here we show that Rab35 controls myelin growth via complex formation with myotubularin-related phosphatidylinositol (PI) 3-phosphatases including MTMR13 and MTMR2 implicated in CMT 4B1 and B2, respectively, to downregulate lipid-mediated mTORC1 activation. Our findings reveal a crucial role for Rab35-regulated lipid turnover by myotubularins in the control of mTORC1 activity and myelin growth suggesting possible avenues for the treatment of CMT 4B-type neuropathies in humans.

## Results

### Rab35•GTP recruits MTMR13-based lipid phosphatase complexes

**Fig. 1:**
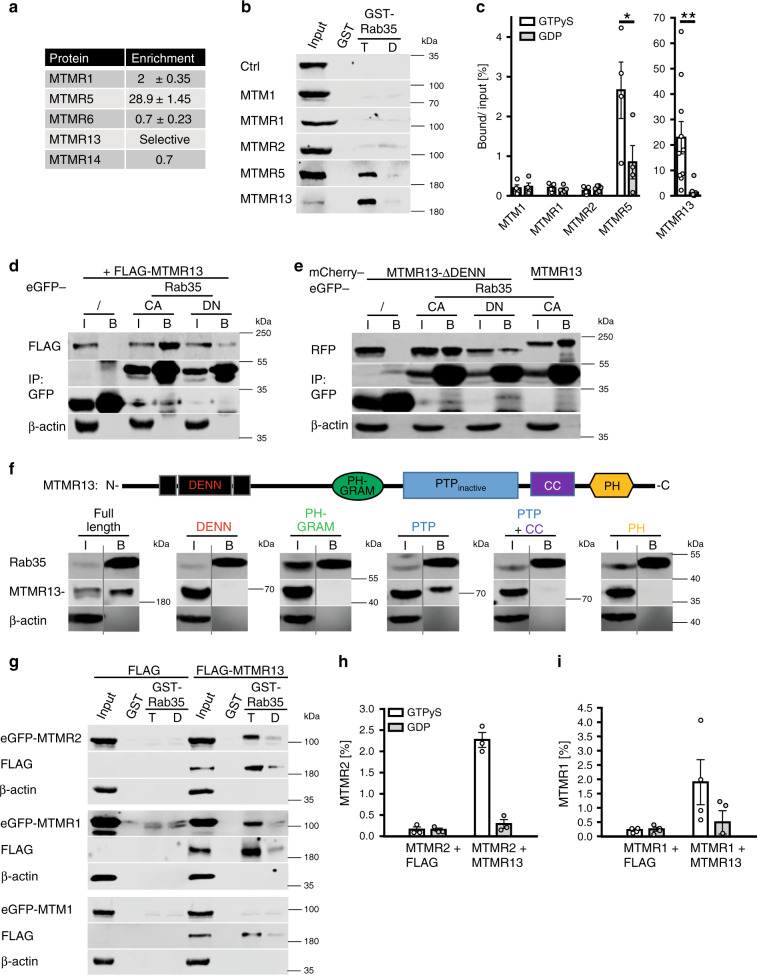
Complex formation of Rab35-GTP with active MTMR lipid phosphatases. **a** BioID screen for Rab35 interacting proteins. Enrichment (BirA*-Rab35/BirA* LFQ ratio) of MTMR protein-derived peptides from two independent experiments (mean ± SD). **b**, **c** Pulldown from lysates of HEK293T cells overexpressing eGFP (ctrl) or the indicated eGFP-tagged MTMRs with immobilized GST-Rab35•GTPγS (T) or GDP (D). **b** Representative immunoblots for eGFP or FLAG (MTMR13). Input: 5 % (MTM1, MTMR1, MTMR2, MTMR5), 6 % (ctrl), 3 % (MTMR13) of total protein added. **c** Quantification of representative data in **b**. Bound protein/input is shown. Two-tailed paired Student’s *t*-test of proteins bound to Rab35•GTP vs. GDP (*n* = independent experiments): MTM1: *n* = 4, *p* = 0.0892, *t* = 2.48, df = 3; MTMR1: *n* = 5, *p* = 0.2314, *t* = 1.41, df = 4; MTMR2: *n* = 7, *p* = 0.0712, *t* = 2.189, df = 6; MTMR5: *n* = 4, *p = 0.0191, *t* = 4.618, df = 3; MTMR13: *n* = 10, **p = 0.0048, *t* = 3.712, df = 9. **d** Affinity capture of eGFP-Rab35 GTP (CA, constitutively active) or GDP (DN, dominant-negative/inactive) locked mutants from lysates of FLAG-MTMR13-overexpressing HEK293T cells. Immunoblot analysis for eGFP, FLAG, or β-actin of input (I) and bound (B) fractions. Ratio of FLAG-MTMR13 bound to CA/DN is 11.6. IP immunoprecipitation. Input (I): 3.5% of total protein. **e** Same as in d but with MTMR13-ΔDENN. **d**, **e** are representative of two independent experiments. **f** Top, MTMR13 domain organization. Bottom, affinity capture of endogenous eGFP-Rab35 from KI HeLa cells expressing full-length MTMR13 or the indicated MTMR13-domains analyzed by immunoblotting as in **d**. Input (I): 4% (PH-GRAM, PTP), 3% (DENN, PTP + CC), 1.5% (Full length, PH) of total protein. Representative of two independent experiments. **g**–**i** Pulldown from lysates of HEK293T cells co-expressing eGFP-MTM1, -MTMR1, or -MTMR2, and FLAG-MTMR13 or FLAG using immobilized active GST-Rab35•GTP. **g** Representative immunoblot as in **d**. Input: 5% of total protein. **h**, **i** Quantification of representative data in g. Bound protein/input is shown. *n* = 3 (MTMR2) or 4 independent experiments. Data are mean ± SEM. See Source Data file for numerical source data and unprocessed blots.

**Fig. 2:**
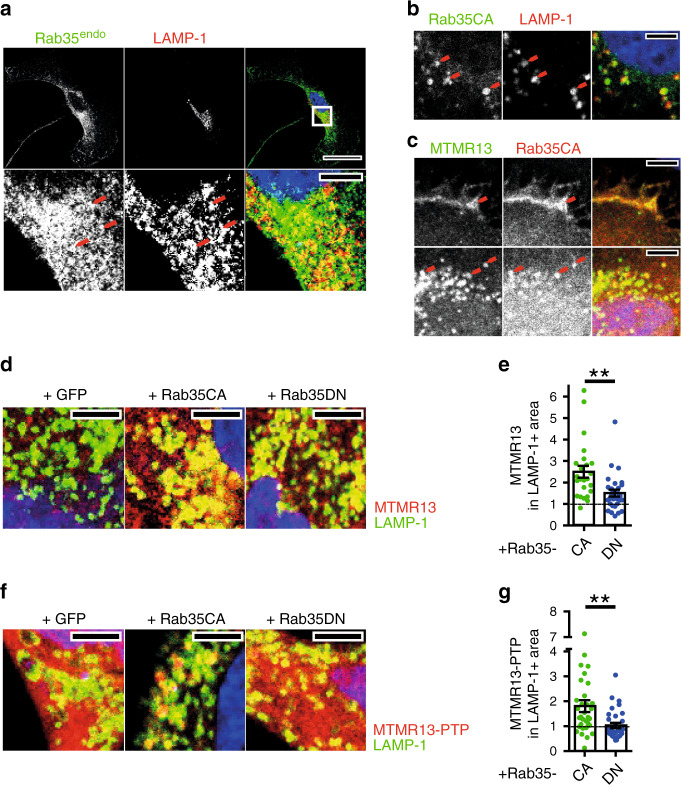
Rab35 can recruit MTMR13 to late endosomes/lysosomes. **a** Partial localization of endogenous eGFP-Rab35^endo^ (green) at lysosomes (LAMP1, red) HeLa cells. Top, white square delineates magnified area (bottom). Red arrows, colocalizing puncta. DAPI (blue), cell nuclei. Scale bars, 30 μm (top), 5 µm (bottom). Representative of 14 cells. **b**, **c** Representative confocal images of HeLa cells expressing constitutively active eGFP-Rab35 (Rab35CA) and FLAG- MTMR13 alone or combined and immunostained for eGFP, LAMP-1, and FLAG. DAPI (blue), cell nuclei. Red arrows, colocalizing puncta. Scale bars, 5 µm. **b** eGFP-Rab35•GTP (Rab35CA) (green) localizes to perinuclear LAMP-1 (red)-positive puncta. Representative of two independent experiments. **c** MTMR13 (green) colocalizes with eGFP-Rab35•GTP (Rab35CA) (red) at the plasma membrane (top) and in perinuclear puncta (bottom). **d**, **e** MTMR13 is cytosolic but localizes to late endosomes/lysosomes in the presence of active Rab35•GTP. *n* = 5 independent experiments. **d** Representative images of HeLa cells co-expressing FLAG-MTMR13 with eGFP, constitutively active eGFP-Rab35•GTP (Rab35CA) or dominant-negative eGFP-Rab35•GDP (Rab35DN). Cells were immunostained for eGFP (see Supplementary Fig. 2c), LAMP-1 (green) and FLAG (red). DAPI (blue), cell nuclei. Scale bars, 5 µm. **e** Quantification of representative images shown in **d**. Mean intensity of MTMR13 in LAMP1-puncta was normalized to MTMR13-expression and set to 1 under control conditions (+eGFP). Data are from *n* = 24 (Rab35CA) and 29 (Rab35DN) cells in four independent experiments. Two-tailed Student’s *t*-test with a theoretical mean of 1, **p = 0.0022, *t* = 3.227, df = 51. **f**, **g** Representative images of HeLa cells co-expressing mCherry-MTMR13-PTP with eGFP, eGFP-Rab35•GTP (Rab35CA), or eGFP-Rab35•GDP (Rab35DN) (**f**). Cells were immunostained for eGFP (see Supplementary Fig. 2d), LAMP-1 (green) and mCherry (red). DAPI (blue), cell nuclei. Scale bars, 5 µm. **g** Quantification (as described in **e**) of representative images shown in **f**. Data are from *n* = 32 (Rab35CA) and 31 (Rab35DN) cells in four independent experiments. Two-tailed Student’s *t*-test with a theoretical mean of 1, **p = 0.0044, *t* = 2.957, df = 61. All data represent mean ± SEM. Numerical source data are reported in the Source Data file.

While Rab35 has been implicated in a multitude of cell physiological functions^20,21^, we know comparably little about the precise molecular mechanisms and protein effectors, e.g. proteins associated with active Rab35-GTP, that underly these roles. To fill this gap, we conducted a non-biased proteomic screen for Rab35 interacting proteins based on BioID^39^, a technique that harnesses the ability of a promiscuous biotin ligase to biotinylate proteins in its close proximity. We expressed a chimeric protein comprised of Rab35 fused to a mutant version of the bacterial BirA* biotin ligase in biotin-fed HEK293T cells (Fig. S1a) and analyzed affinity-purified biotinylated proteins co-enriched with Rab35-BirA* over BirA* by quantitative mass spectrometry (Supplementary Table 1 and Supplementary Data 1). This analysis revealed a striking association of Rab35 with MTMR13 (Supplementary Data 1) and MTMR5 (Supplementary Table 1), two myotubularin-related catalytically inactive PI 3-phosphatases implicated in myelin biogenesis and CMT in humans (Fig. 1a). Affinity chromatography experiments using GST-Rab35•GTP (active) or -Rab35•GDP (inactive) as baits confirmed specific binding of MTMR13 and, less efficiently, MTMR5 to Rab35•GTP in vitro (Fig. 1b, c). A similar preference of MTMR13 for active Rab35•GTP was observed in co-immunoprecipitation experiments employing GDP- vs. GTP-locked variants of GFP-Rab35 and FLAG-MTMR13 co-expressed in HEK293T cells (Fig. 1d). Complex formation between Rab35 and MTMR13 or MTMR5 (consistent with data from *Drosophila*^40^) was specific as other myotubularin family members such as MTM1, MTMR1, or MTMR2 when expressed on their own failed to bind to GST-Rab35•GTP (Fig. 1b, c). As Rab35•GTP associated with MTMR13 with preference over MTMR5 we focussed on the Rab35/MTMR13 interaction. First, we observed that MTMR13 associates with Rab35 but not with GST-fused Rab1A, Rab5, Rab7, or Rab11 irrespective of their nucleotide loading status (Supplementary Fig. 1b). Second, we made use of HeLa knockin (KI) cells expressing GFP-Rab35 from its endogenous locus^30^.We found that GFP-Rab35^endo^ captured on GFP-nanotrap beads co-purified with mCherry-MTMR13 expressed in these cells (Fig. 1f). Further biochemical mapping experiments revealed that MTMR13 complex formation with Rab35 was mediated, in part, by its catalytically inactive PTP phosphatase domain (Fig. 1f), although other domains may also contribute to binding. Consistently, a mutant of MTMR13 lacking its DENN domain (and, hence, devoid of its putative GEF activity), associated with constitutively active GFP-Rab35•GTP but not with dominant-negative GFP-Rab35•GDP in transfected HEK293cells (Fig. 1e). These data suggest that Rab35•GTP associates with myotubularin family proteins including MTMR13 possibly to aid its recruitment to intracellular membranes. As no antibodies suitable for detection of endogenous MTMR13 are available, we probed this possibility by analyzing the subcellular distribution of overexpressed MTMR13 in transfected HeLa cells. When expressed on its own MTMR13 exhibited a largely cytoplasmic distribution (Supplementary Fig. 2c). Endogenous GFP-Rab35^endo^ in addition to its known localization to peripheral endosomes and the plasma membrane partially colocalized with the late endosomal/lysosomal marker LAMP-1 (Fig. 2a), a localization pattern that was even more pronouncedly observed for GTP-locked Rab35 (Rab35 CA) (Fig. 2b, Supplementary Fig. 2a). Interestingly, expression of constitutively active mutant Rab35 sufficed to induce the recruitment of co-expressed MTMR13 to the cell surface (Fig. 2c, Supplementary Fig. 2b) and to LAMP1-positive late endosomes/lysosomes, while inactive GDP-locked Rab35 was less potent (Fig. 2d, e; Supplementary Fig. 2c). Active Rab35-mediated recruitment to LAMP1-positive late endosomes/lysosomes was also seen for the PTP domain of MTMR13 (Fig. 2f,g; Supplementary Fig. 2d), further confirming our biochemical mapping results (Fig. 1f). Rab35•GTP, thus, can recruit MTMR13 to intracellular membranes including late endosomes or lysosomes.

Although MTMR13 is catalytically inactive, it is known to form tetrameric assemblies with MTMR2 (another CMT gene) and, likely, the closely related MTMR1 resulting in lipid phosphatase complexes that hydrolyze phosphatidylinositol 3-phosphate [PI(3)P] and phosphatidylinositol 3,5-bisphosphate [PI(3,5)P_2_]^9^. We therefore probed whether Rab35•GTP may bind lipid phosphatase complexes comprising MTMR13 with active MTMR2 or MTMR1. Indeed, GST-Rab35•GTP efficiently captured MTMR2 or MTMR1, but not MTM1, when co-expressed with MTMR13 (Fig. 1g–i; Supplementary Fig. 1c) but not when expressed alone. Qualitatively similar results were seen for MTMR5-based lipid phosphatase complexes (Supplementary Fig. 1d–f). Hence, Rab35•GTP can bind and recruit MTMR13 and, less efficiently, MTMR5-based active lipid phosphatase complexes.

Collectively, these findings uncover Rab35 as an interactor of MTMR13-based lipid phosphatase complexes.

### Rab35 loss in Schwann cells causes focal hypermyelination

**Fig. 3:**
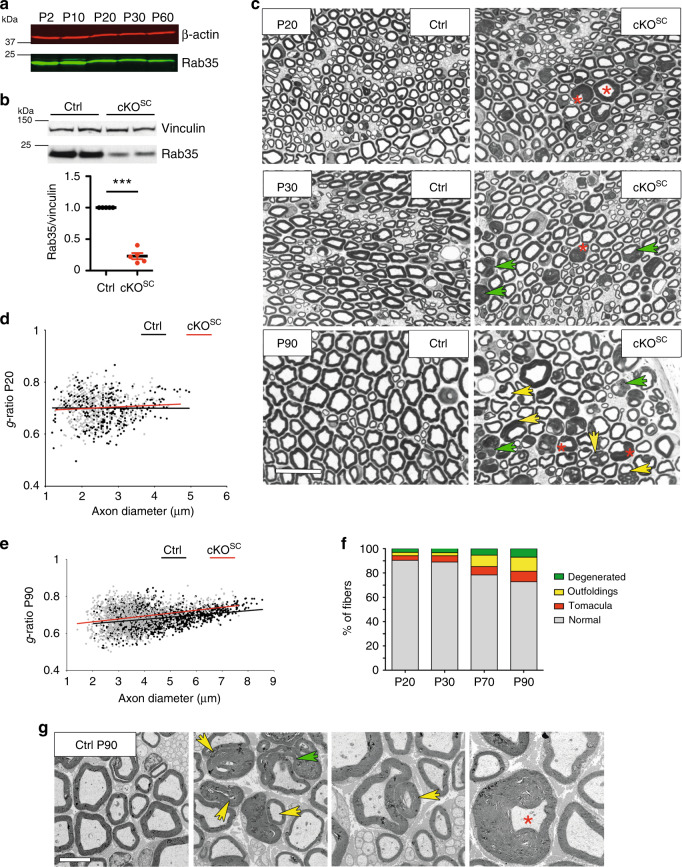
Loss of Rab35 in Schwann cells causes focal hypermyelination. **a** Postnatal Rab35 expression in sciatic nerves. Represenative of two independent experiments. **b** Reduced Rab35 levels in sciatic nerve lysates from *Rab35* cKO^SC^ mice at P30 compared to controls (Ctrl) set to 1. One-sample two-sided *t*-test, *n* = 5 pools of nerves per genotype in three independent experiments, *p* = 0.0001, *t* = 15.52, df = 4. Residual Rab35 is from fibroblasts and axons. **c** Semithin section analysis of control and *Rab35* cKO^SC^ sciatic nerves at P20, P30, and P90. Red asterisks, examples of tomacula or increased myelin thickness. Yellow arrowheads, myelin outfoldings. Green arrowheads, myelin degenerations. Representative images for quantifications in **f**. **d** g-ratio at P20: *Rab35* cKO^SC^, 0.7 ± 0.005, 404 fibers; controls (*Rab35*^*flox/flox*^ and *Rab35*^*flox/+*^), 0.7 ± 0.0024, 432 fibers, *n* = 5 mice/genotype, *p* = 0.69. **e** g-ratio at P90: *Rab35* cKO^SC^, 0.688 ± 0.002, 1162 fibers; controls (*Rab35*^*flox/+*^), 0.682 ± , 0.002, 1499 fibers, *n* = 4 mice, *p* = 0.057. Fibers >5 µm in diameter: *Rab35* cKO^SC^, 0.73271374 ± 0.005, 130 fibers and control (*Rab35*^*flox/+*^), 0.700473046 ± 0.0045, 446 fibers, *n* = 4 mice, *p* = 0.0286. **d**, **e** Two-tailed nonparametric Mann–Whitney test. **f** Myelinated fibers with aberrant myelin (in percentage of total): *Rab35* cKO^SC^ at P20, *n* = 5 mice, percentage of Myelin degeneration: 3.053 ± 0.272. Tomacula: 3.684 ± 0.164. Myelin outfoldings: 2.829 ± 0.313; at P30, *n* = 4 mice, Myelin degeneration: 3.348 ± 0.221. Tomacula: 5.109 ± 0.508. Myelin outfoldings: 2.548 ± 0.136; at P70, *n* = 7 mice. Myelin degeneration: 5.390 ± 0.472. Tomacula: 6.916 ± 0.389. Myelin outfoldings: 9.336 ± 1.008, and at P90, *n* = 4 mice. Myelin degeneration: 6.993 ± 0.572; tomacula: 8.553 ± 0.676. Myelin outfoldings: 11.619 ± 0.588. **g** Representative images of ultrastructural analysis of control and *Rab35* cKO^SC^ sciatic nerves (middle, right) at P90 quantified in **f**. Red asterisk, tomacula. Yellow arrowheads, myelin outfoldings. Green arrowhead, example of myelin degeneration. Mean ± SEM. Bar in **c** for P20, P30, and P90 semithin sections is 20 µm. Bar in **g** is 5 µm and 3.5 µm (for the outfolding and tomacula details). Numerical source data and unprocessed blots are reported in the Source Data file.

Given the prominent genetic and functional association of MTMR13 and its catalytic subunit MTMR2 with CMT and demyelinating neuropathies in humans and in defective myelin biogenesis in mice^2,4,41–43^, we hypothesized that Rab35 may regulate myelin biogenesis in vivo. Consistent with this hypothesis, we found Rab35 to be highly expressed in sciatic nerve lysates from newborn mice at P2, i.e. around the onset of myelination, whereas its expression levels declined during postnatal PNS development (Fig. 3a). To explore the possible function of Rab35 in myelin biogenesis in the PNS, we targeted the *Rab35* locus for genetic manipulation by introducing *lox*P recombination sites that allow for the conditional disruption of Rab35 expression (Supplementary Fig. 3). Constitutive disruption of the *Rab35* locus in β-actin Cre expressing strains resulted in embryonic lethality. To bypass this, we crossed *Rab35*^*flox/flox*^ mice with a transgenic mouse line expressing Cre recombinase under the control of the myelin protein zero (*P0*) promoter resulting in the Schwann cell (SC)-specific downregulation of Rab35 expression as early as E13.5^44^. *Rab35*^*flox/flox*^ *P0-Cre* mice (hereafter termed cKO^SC^) were born according to a Mendelian ratio and did not display any evident clinical phenotype. Sciatic nerve lysates from *Rab35* cKO^SC^ showed a strong reduction of Rab35 protein (Fig. 3b; the remaining Rab35 signal likely originates from axons and/or fibroblasts present in the sciatic nerve). Ultrastructural or semithin section analyses from their sciatic nerves at postnatal day 20 (P20) (Fig. 3c, d), P30 and P90 (Fig. 3c, e) revealed a normal number of myelinated axons and unaltered myelin thickness. Only at P90 large fibers (>5 µm in diameter) displayed reduced myelin thickness (Fig. 3e). Interestingly, myelinated fibers from *Rab35* cKO^SC^ sciatic nerves showed abundant signs of focal hypermyelination including tomacula and myelin outfoldings, in addition to myelin degeneration (Fig. 3f, g). These myelin abnormalities, which were never observed in control nerves (Fig. 3c, g) progressively worsened with the age of the animals (Fig. 3c, f). These findings indicate that loss of Rab35 expression in Schwann cells recapitulates morphological defects in myelin architecture observed in other IPN/CMT models including loss of MTMR2 and MTMR13 in mice^42,45^.  Our data reveal a prominent role for Rab35 in the control of myelin growth that is consistent with a physical and functional interaction with the CMT-associated lipid phosphatases MTMR13 and MTMR2.

### Rab35 and its associated MTMRs repress mTORC1 activity

**Fig. 4:**
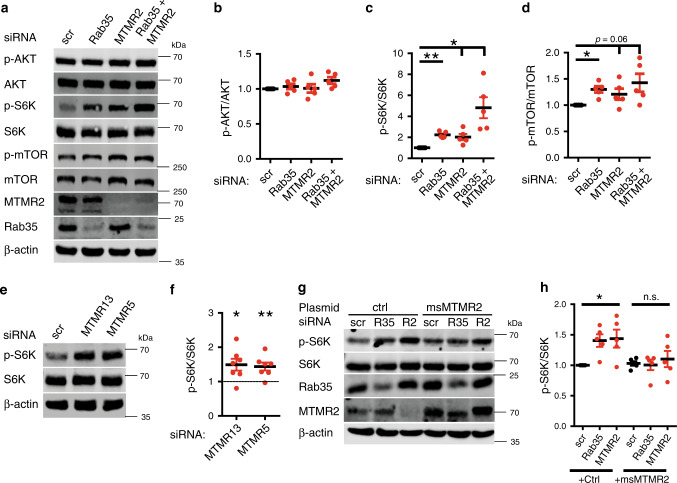
Rab35 or MTMR2 depletion results in mTORC1 hyperactivation. **a** Representative immunoblot of HEK293T cells depleted of Rab35, MTMR2, or both. **b**–**d** Quantification of representative data in **a** from *n* = 5 independent experiments. All normalized to scrambled (scr). **b** p-AKT/AKT ratio: Rab35 siRNA (Rab35): *p* = 0.93502, *t* = 0.9335, df = 4; MTMR2 siRNA (MTMR2): *p* = 0.9073, *t* = 0.12404, df = 4; Rab35 siRNA + MTMR2 siRNA (Rab35+MTMR2): *p* = 0.18496, *t* = 2.57498, df = 4. **c** p-S6K/total S6K ratio: Rab35 siRNA (Rab35): **p = 0.0023, *t* = 9.2329, df = 4; MTMR2 siRNA (MTMR2): *p = 0.0374, *t* = 3.43714, df = 4; Rab35 siRNA+MTMR2 siRNA (Rab35+MTMR2): *p = 0.0374, t = 3.8251, df = 4. **d** p-mTOR/total mTOR ratio: Rab35 siRNA (Rab35): *p = 0.0116, *t* = 4.95093, df = 4; MTMR2 siRNA (MTMR2): *p* = 0.0688, *t* = 1.99438, df = 4; Rab35 siRNA+MTMR2 siRNA (Rab35+MTMR2): *p* = 0.0688, *t* = 2.47145, df = 4. **e**, **f** Immunoblot analysis of HEK293T cells depleted of MTMR13 or MTMR5. **e** Representative immunoblot for p-S6K, total S6K. **f** Quantification of representative data in **e** from *n* = 7 independent experiments. Normalized p-S6K/total S6K ratio (scr = 1). One sample two-tailed Student’s *t*-test (theoretical mean of 1): MTMR13 siRNA (MTMR13): *p = 0.0293, *t* = 2.846, df = 6; MTMR5 siRNA (MTMR5): **p = 0.0097, *t* = 3.733, df = 6. **g**, **h** Immunoblot analysis of Rab35- or MTMR2-depleted HEK293T cells expressing eGFP (ctrl) or siRNA-resistant mouse MTMR2 (msMTMR2). **g** Representative immunoblot. **h** Normalized p-S6K/total S6K ratio from *n* = 5 independent experiments. ctrl: Rab35 siRNA: *p = 0.0338, *t* = 3.94437, df = 4; MTMR2 siRNA: *p = 0.0416, *t* = 2.95823, df = 4; msMTMR2: Rab35 siRNA: *p* = 0.8, *t* = 0.27059, df = 4, MTMR2 siRNA: *p* = 0.59186, *t* = 0.58188, df = 4. n.s., non-significant. Data represent mean±SEM. One sample two-tailed Student’s *t*-test (theoretical mean of 1) followed by Holm’s Multiple Comparison Test for quantifications in (**b**–**d**, **h**). See Source Data file for numerical source data and unprocessed blots.

Focal hypermyelination including tomacula and myelin outfoldings have been found to be associated with hyperactive mTORC1 signaling, for example as a consequence of genetic inactivation of the mTORC1 repressor TSC1 or the lipid phosphatase PTEN, a major negative regulator of the PI 3-kinase-AKT-mTORC1 pathway^14,46,47^. Based on these results and the fact that the MTMR substrates PI(3)P and PI(3,5)P_2_ can activate mTORC1 signaling^10–12^, we hypothesized that Rab35 via complex formation with MTMR-based lipid phosphatase complexes at late endosomes/lysosomes may locally repress mTORC1 activity. We therefore analyzed mTORC1 signaling in HEK293T cells depleted of endogenous Rab35 and/or MTMR2. Depletion of either Rab35 or MTMR2 caused a significant elevation in the steady-state levels of phospho-mTOR/total mTOR, or phospho-S6 kinase 1 (p-S6K)/total S6K levels, indicative of mTORC1 hyperactivity (Fig. 4a, c, d). A similar increase in mTORC1 activity was observed upon knockdown of either MTMR5 or MTMR13 (Fig. 4e, f; Supplementary Fig. 4a, b). Co-depletion of Rab35 and MTMR2 led to an even more robust increase in mTORC1 signaling (Fig. 4a, c, d), in line with the fact that Rab35 can associate with MTMR13-MTMR2 and other lipid phosphatase complexes including MTMR5 and MTMR1 (compare Fig. 1, Supplementary Fig. 1). In contrast, loss of Rab35 and/or MTMR2 in HEK293 cells did not result in significant alterations in AKT signaling (Fig. 4b) consistent with previous data on MTMR2 in Schwann cells^48^. These data suggest that Rab35 and its associated MTMRs may regulate mTORC1 activity independent of AKT.

**Fig. 5:**
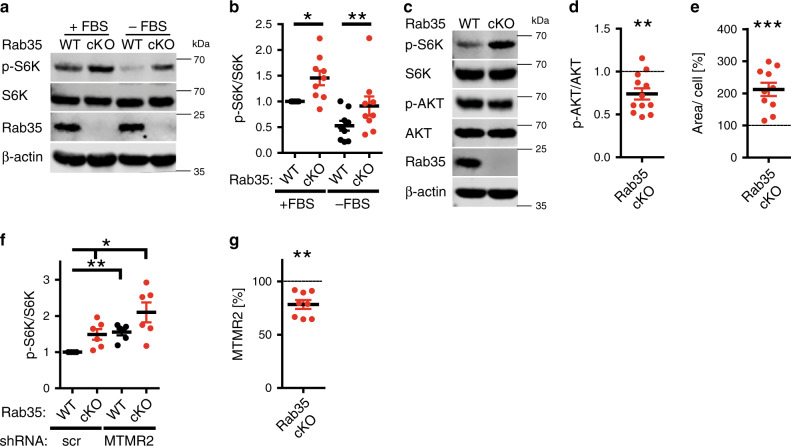
Acute Rab35 depletion causes mTORC1 hyperactivation. Astrocytes from tamoxifen-inducible *Rab35* cKO (*Rab35*^*flox/flox*^; *CAG-Cre*^*ER*^) or non-Cre^ER^-expressing control mice (*Rab35*^*flox/flox*^, WT) were treated with tamoxifen and serum-fed (+FBS) or starved (−FBS) as indicated. **a** Representative immunoblot for p-S6K, total S6K, and ß-actin. **b** p-S6K/total S6K ratio in WT (set to 1) and cKO astrocytes. *n* = 9 independent experiments. One sample two-tailed Student’s *t*-test with a theoretical mean of 1; +FBS: *p = 0.0124, *t* = 3.211, df = 8; −FBS: **p = 0.0093, *t* = 3.404, df = 8. **c**, **d** Representative immunoblot of WT and *Rab35* cKO astrocyte lysates probed for p-S6K, total S6K, p-AKT, total AKT, Rab35, and ß-actin (**c**). **d** p-AKT/AKT ratio in WT (set to 1) and cKO astrocytes. *n* = 12 independent experiments. One sample two-tailed Student’s *t*-test with a theoretical mean of 1; **p = 0.0022, *t* = 3.965, df = 11. **e** Area of WT and cKO astrocytes shown in Supplementary Fig. 5g. *n* = 10 independent experiments with WT set to 100. One sample two-tailed Student’s *t*-test with a theoretical mean of 100; ***p = 0.0004, *t* = 5.411, df = 9. **f** p-S6K/total S6K ratio in *Rab35* WT (set to 1) and cKO astrocytes transduced with lentiviruses encoding scrambled (scr) or anti-MTMR2 shRNA from *n* = 6 independent experiments. See Supplementary Fig. 5p. One sample two-tailed Student’s *t*-test with a theoretical mean of 1, for all conditions normalized to control (WT + scr), followed by Holm’s Multiple Comparison Test to correct for multiple testing; cKO + scr: *p = 0.0209, *t* = 3.32454, df = 5; WT + shMTMR2: **p = 0.0047, *t* = 6.21728, df = 5; cKO + shMTMR2: *p = 0.0205, *t* = 4.00827, df = 5. **g** MTMR2 levels in lysates from *Rab35* WT and cKO astrocytes. See representative data in Supplementary Fig. 5s. *n* = 8 independent experiments. One sample two-tailed Student’s *t*-test with a theoretical mean of 100, **p = 0.0013, *t* = 5.17, df = 7. All data represent mean ± SEM. See Source Data file for numerical source data and unprocessed blots.

**Fig. 6:**
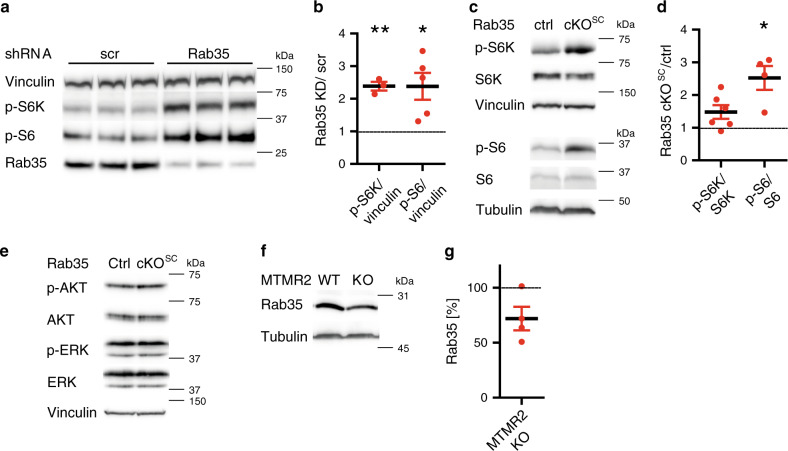
Acute Rab35 depletion results in mTORC1 hyperactivation in vivo. **a**, **b** p-S6 and p-S6K levels in Schwann cell/DRG neuron co-culture explants transduced with scrambled (scr) or *Rab35*-targeting shRNA lentiviruses. **a** Representative immunoblot of lysates from shRNA-transduced co-cultures. **b** Quantification of representative data in a. Scr were set to 1. One sample two-tailed Student’s *t*-test with a theoretical mean of 1: p-S6K/Vinculin: **p = 0.0092, *t* = 10.35, df = 2, *n* = 3 DRG pools of at least 10 explants each; p-S6/Vinculin: *p = 0.0286, *t* = 3.35, df = 4, *n* = 5 DRG pools of at least 10 explants each. **c**–**e** Immunoblot analysis of sciatic nerves from P30 *Rab35* control (*Rab35*^*flox/flox*^) and cKO^SC^ (*Rab35*^*flox/flox*^ *P0-Cre*) mice. **c** Representative immunoblot for p-S6, total S6, p-S6K, and total S6K. Tubulin, Vinculin as loading controls. **d** Quantification of representative data in **c**. Controls were set to 1. One sample two-tailed Student’s *t*-test with a theoretical mean of 1: p-S6/S6: *p = 0.0253, *t* = 1.455, df = 3, *n* = 4 pools of nerves per genotype and independent experiments; p-S6K/S6K: *p* = 0.0706, *t* = 2.291, df = 5, *n* = 6 pools of nerves per genotype from four independent experiments. **e** Representative immunoblot for p-AKT, total AKT, S44/42-phosphorylated ERK (p-ERK), and total ERK in sciatic nerve lysates. Vinculin was analyzed as a loading control. **f**, **g** Rab35 protein levels are decreased in sciatic nerve lysates from *Mtmr2* KO mice. Sciatic nerves from *Mtmr2* WT (*Mtmr2*^*+/+*^) and KO (*Mtmr2*^*−/−*^) mice at P30 were lysed and analyzed by immunoblotting, **f** Representative immunoblot for Rab35, and tubulin as a loading control. **g** Quantification of representative data shown in **f**. Data are from *n* = 4 pools of nerves per genotype from four animals per genotype; One sample two-tailed Student’s *t*-test with a theoretical mean of 1, *p* = 0.0789, *t* = 2.622, df = 3. Data represent mean ± SEM. See Source Data file for numerical source data and unprocessed blots.

To dissect whether genetic loss of Rab35 affects mTORC1 signaling in cells of the nervous system we crossed our *Rab35*^*flox/flox*^ line with *CAG-Cre*^*ER*^ mice allowing for the conditional KO (cKO) of Rab35 in primary astrocytes in culture (Supplementary Fig. 5a) following addition of tamoxifen (Supplementary Fig. 5b). *Rab35* cKO astrocytes displayed significantly elevated levels of p-S6/total S6 (Supplementary Fig. 5c, d) and p-S6K/S6K and these partially persisted following serum starvation, i.e. conditions under which p-AKT is inactive (Fig. 5a, b). Increased mTORC1 activity was not a consequence of overactive p-AKT signaling (Fig. 5c, d), which, instead, was reduced in KO astrocytes (similar to recent data from mouse embryonic fibroblasts^29^ and different from our results in HEK293 cells discussed above). Moreover, *Rab35* cKO astrocytes were significantly larger than their control (*Rab35*^*flox/flox*^ without Cre) counterparts (Fig. 5e, Supplementary Fig. 5g), consistent with the established function of mTORC1 in regulating cell size^14,19^. Surprisingly, cKO of Rab35 did not cause any overt defects in the endolysosomal system as evidenced by unaltered levels and appearance of EEA1-containing early endosomes, Rab7-positive late endosomes, or lysosomes probed by LAMP-1 and LAMP-2 (Supplementary Fig. 5h–o). We did, however, notice a mild reduction in the expression of the autophagy marker LC3, possibly as a consequence of reduced TFEB-mediated expression of autophagy genes under conditions of elevated mTORC1 activity (Supplementary Fig. 5e, f). mTORC1 hyperactivity in *Rab35* cKO astrocytes was further aggravated by lentiviral co-depletion of MTMR2 (Fig. 5f; Supplementary Fig. 5p, r), akin to results from HEK293T cells, whereas the observed reduction of p-AKT signaling in *Rab35* cKO astrocytes was unaffected by MTMR2 loss (Supplementary Fig. 5q). Similar changes in nutrient signaling via mTORC1 occurred in Schwann cell/DRG neuron co-cultures acutely depleted of Rab35 by lentiviral knockdown (Fig. 6a, b) and in sciatic nerve lysates from Schwann cell-specific cKO mice (*Rab35* cKO^SC^) that display focal hypermyelination (Fig. 6c, d). In contrast, the steady-state levels of p-ERK or p-AKT were unchanged in nerve lysates from cKO mice (Fig. 6e). These collective data suggest that Rab35 via complex formation with MTMR-based lipid phosphatase complexes represses mTORC1 signaling. This model is further supported by the observations that (i) MTMR2 levels are significantly reduced in *Rab35* cKO astrocytes (Fig. 5g, Supplementary Fig. 5s), while conversely, (ii) expression of Rab35 was decreased in sciatic nerve lysates from *Mtmr2* KO mice (Fig. 6f, g), consistent with the idea that Rab35 and MTMR2 operate in a complex. Moreover, (iii) overexpression of active MTMR2 reduced mTORC1 activity to near normal levels in cells depleted of Rab35, MTMR2 (Fig. 4g, h), or MTMR13 (Supplementary Fig. 4c). In contrast, inactive MTMR2 was less effective at rescuing elevated mTORC1 activity in cells depleted of MTMR13 (Supplementary Fig. 4c) or Rab35 (Supplementary Fig. 4d).

**Fig. 7:**
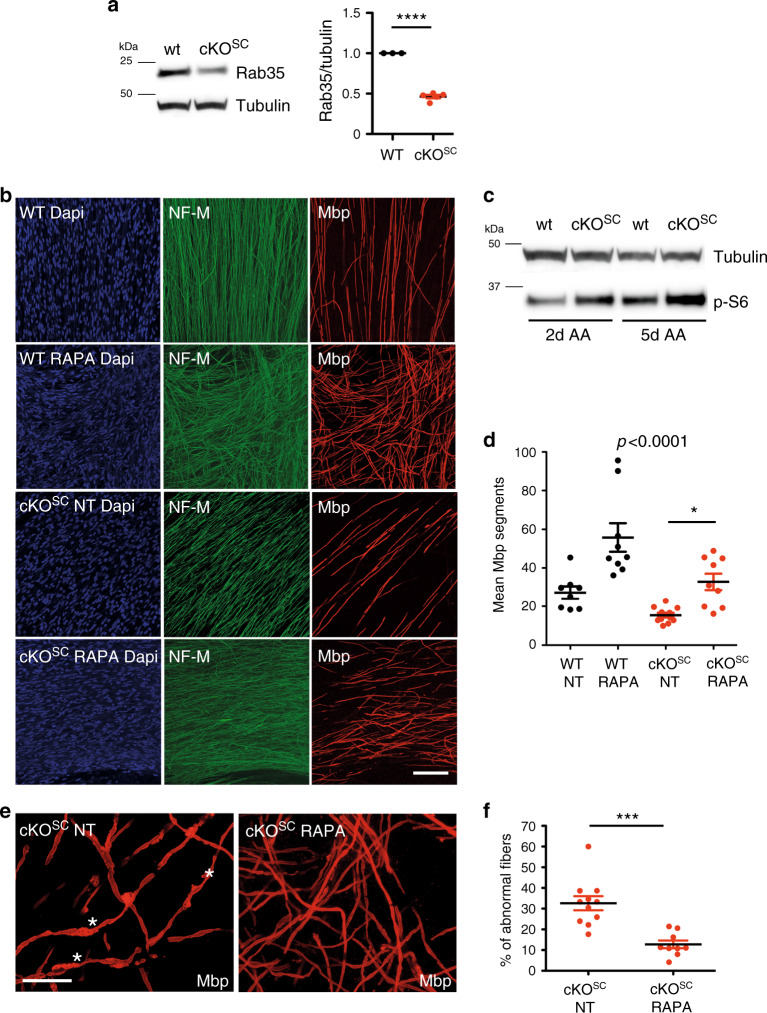
mTORC1 inhibition ameliorates hypermyelination caused by loss of Rab35. **a** Loss of Rab35 protein expression in Schwann cell/DRG neuron co-culture explants from *Rab35* WT and cKO^SC^ mouse embryos, One sample two-tailed Student’s *t*-test with a theoretical mean of 1, *p* < 0.0001, *t* = 25, df = 4, *n* = 3 WT, and *n* = 5 cKO^SC^ DRG pools of at least 10 explants each. **b** Representative confocal images of myelin-forming Schwann cell/DRG neuron co-culture explants from *Rab35* WT, wild-type (*Rab35*^*flox/flox*^) and cKO^SC^ mouse embryos either left untreated (NT) or treated with Rapamycin (RAPA). DAPI staining shows Schwann cell nuclei; NF-M staining, neurofilament, and Mbp, myelin basic protein to detect myelin segments. **c** Levels of p-S6 in WT and cKO^SC^ DRG explants following 2 and 5 days of ascorbic acid treatment in differentiating conditions. *n* = 1 independent experiment. **d** Quantification of the number of Mbp-positive myelin segments in co-culture explants from *Rab35* WT and cKO^SC^ embryos either left untreated (NT) or treated with Rapamycin (RAPA): WT (NT): 27.18 ± 3.22, *n* = 8 DRG/coverslips; WT (RAPA): 54.51 ± 7.641, *n* = 9 DRG/coverslips; cKO^SC^ (NT): 15.38 ± 1.2, *n* = 11 DRG/coverslips; cKO^SC^ (RAPA): 32.79 ± 4.26, *n* = 9 DRG/coverslips, one-way non-parametric ANOVA followed by Dunn’s Multiple Comparison Test, *p* < 0.0001. **e** Representative confocal images and **f** quantification of the fraction of Mbp-positive fibers carrying myelin abnormalities in *Rab35* cKO^SC^ explants either left untreated (NT) or treated with Rapamycin (RAPA). Asterisks mark Mbp-positive fibers carrying abnormalities. cKO^SC^ (NT): 32.62 ± 3.42, *n* = 330 fibers in *n* = 11 coverslips/DRG explants; cKO^SC^ (RAPA): 12.73 ± 1.89, *n* = 311 fibers in *n* = 9 coverslips/DRG explants, ***p = 0.0004, two-tailed nonparametric Mann–Whitney test. Data represent mean ± SEM. Bar in **b** is 91 µm and in **e** 35 µm. See Source Data file for numerical source data and unprocessed blots.

If mTORC1 hyperactivity in the absence of Rab35 causally contributes to focal hypermyelination found in Schwann cell-specific *Rab35* cKO^SC^ mice this phenotype might be ameliorated by pharmacological modulation of mTORC1 signaling. To test this, we analyzed the effects of mTORC1 suppression induced by application of the specific mTORC1 inhibitor Rapamycin in myelin-forming Schwann cell/DRG neuron co-culture explants established from “WT” control (*Rab35*^*flox/flox*^ genotype) and *Rab35* cKO^SC^ mouse embryos (Fig. 7a). mTORC1 signaling plays a developmental timing-dependent dual role in peripheral nerve myelination: hyperactive mTORC1 during early nerve development delays the onset of myelination by suppressing differentiation^16^, while mTORC1 hyperactivity in differentiated myelinating Schwann cells causes focal hypermyelination^14,46^. Consistent with this, we observed a significantly reduced number of myelin segments marked by myelin basic protein (Mbp) in co-cultures from *Rab35* cKO^SC^ embryos compared to control (Fig. 7b), likely as a consequence of overactivated mTORC1 signaling in these explants particularly at the onset of myelination between two and seven days of ascorbic acid treatment in differentiating conditions (Fig. 7c). Those fibers that had undergone myelination displayed aberrant myelin (similar to our observations in vivo, compare Fig. 3) with a complex shape and, in part, resembling myelin outfoldings of *Mtmr2* KO cultures (Fig. 7e). Importantly, treatment of co-culture explants from *Rab35* cKO^SC^ embryos with Rapamycin rescued impaired myelin segment formation and ameliorated aberrant myelin (Fig. 7d–f)

**Fig. 8:**
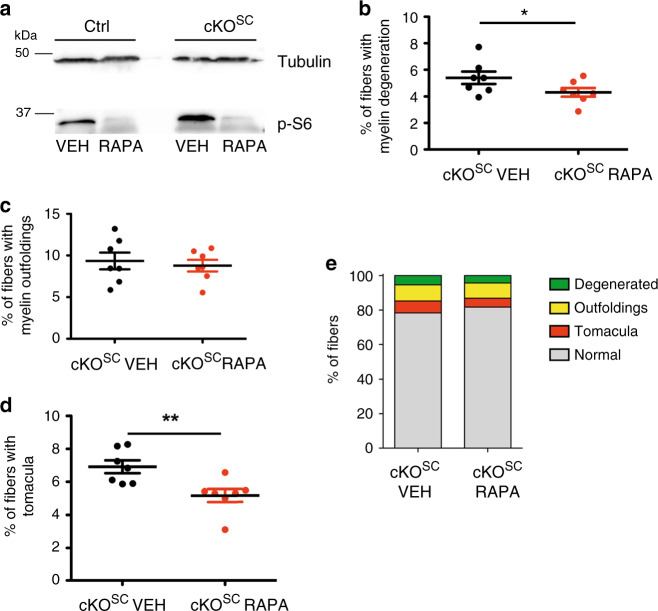
Repression of mTORC1 hyperactivity ameliorates focal hypermyelination in vivo. **a** Rapamycin represses mTORC1 signaling in sciatic nerves in vivo. Immunoblot for p-S6 in sciatic nerve lysates from *Rab35* control and *Rab35* cKO^SC^ mice treated (from P12 until P70) with vehicle or Rapamycin. **b**–**d** Semithin section analysis of sciatic nerves from *Rab35* cKO^SC^ mice (P70) following vehicle or Rapamycin treatment and quantification of the percentage of fibers carrying myelin degeneration. Data represent mean ± SEM. **b**: 5.39% ± 0.47 vehicle-treated, 4.31% ± 0.33 Rapamycin-treated, one-tailed non-parametric Mann**–**Whitney *t*-test, *p = 0.048; myelin outfoldings **c**: 9.33% ± 1.00 vehicle-treated, 8.78% ± 0.70 Rapamycin-treated, one-tailed non-parametric Mann–Whitney *t*-test, *p* = 0.45; and tomacula **d**: vehicle-treated, 6.92% ± 0.39 and Rapamycin-treated, 5.18% ± 0.39, two-tailed nonparametric Mann–Whitney, **p = 0.0041. **e** Quantification of the number of myelinated fibers carrying aberrant myelin in *Rab35* cKO^SC^ vehicle and Rapamycin-treated sciatic nerves. Altered fibers are expressed as a percentage of the total number of fibers. Data are from *n* = 7 mice from each genotype. Data represent mean ± SEM. Source Data file for numerical source data and unprocessed blots.

To corroborate these findings in vivo, we treated *Rab35* cKO^SC^ mutants and littermate controls (*Rab35*^*flox/flox*^) with Rapamycin. The drug was given from P12, when mice can still tolerate long-term Rapamycin treatment, to age P70, when the clinical phenotype was pronounced. Immunoblotting of sciatic nerve lysates of Rapamycin-treated control or *Rab35* cKO^SC^ animals displayed strongly diminished p-S6 levels compared to placebo-treated controls, demonstrating efficient blockage of the mTORC1 pathway (Fig. 8a). Rapamycin treatment significantly reduced aberrant myelin, particularly fibers carrying tomacula (Fig. 8d, e) and, to a lesser extent, myelin degeneration (Fig. 8b), while no significant effect on outfoldings was observed (Fig. 8c), possibly as a result of the very early onset of the phenotype (Supplementary Fig. 6c). Of note, at either P3 or P5, prior to Rapamycin treatment, we observed a consistent number of altered myelinated fibers carrying myelin degeneration or focal hypermyelination (Supplementary Fig. 6b, e) with a similar number of myelinated fibers (Supplementary Fig. 6a, d) and of myelin thickness between control and *Rab35* cKO^SC^ sciatic nerves (g-ratio as a function of axonal diameter in sciatic nerves at P3: *Rab35* cKO^SC^, 0.8245 ± 0.0059, 192 fibers; controls (*Rab35*^*flox/+*^), 0.834 ± , 0.011, 194 fibers, *n* = 3 animals per genotype, *p* = 0.7000. P5: *Rab35* cKO^SC^, 0.7542 ± 0.0052, 443 fibers; controls (*Rab35*^*flox/+*^), 0.749 ± , 0.0085, 527 fibers, *n* = 5 animals per genotype, *p* = 0.6905). These findings suggest that elevation of mTORC1 signaling does not impair the onset of myelination and is consistent with the observed extent of mTORC1 overactivity at P30 (compare Fig. 6c, d). Collectively, our data are consistent with the hypothesis that elevated mTORC1 activity in the absence of Rab35 contributes to focal hypermyelination in *Rab35* cKO^SC^ mice, a phenotype that is ameliorated by pharmacological inhibition of mTORC1 signaling by Rapamycin.

### Rab35 and MTMRs regulate mTORC1 by PI 3-phosphate hydrolysis

**Fig. 9:**
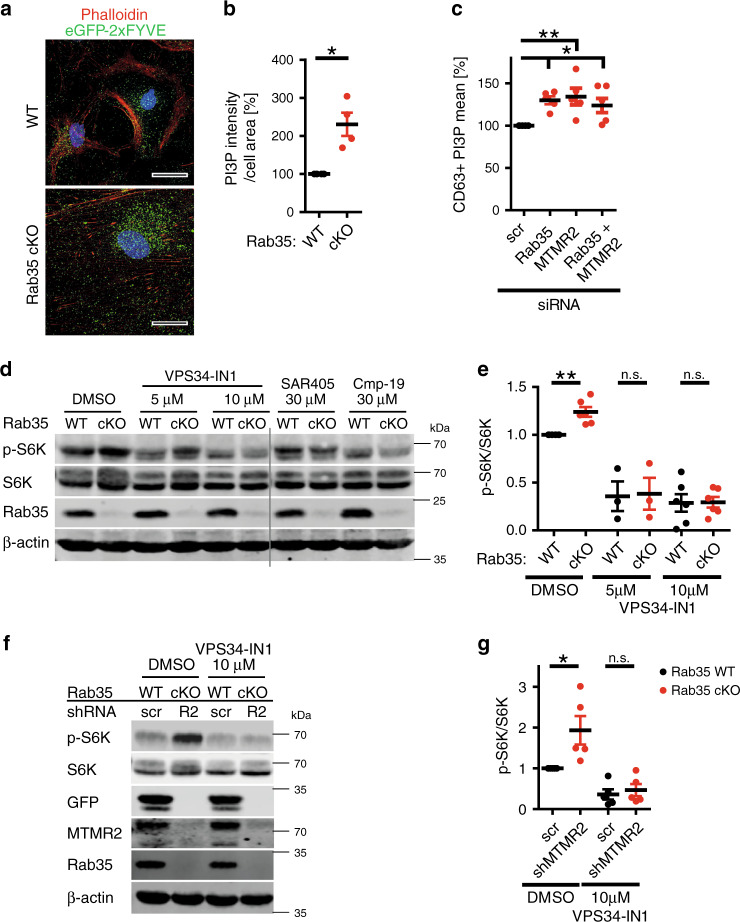
Inhibition of PI 3-phosphate synthesis rescues mTORC1 hyperactivity. **a** Representative images of astrocytes from *Rab35*^*flox/flox*^ cKO or non-Cre^ER^-expressing *Rab35*^*flox/flox*^ control mice (WT) stained for PI(3)P (green) and F-actin (red). Blue, DAPI-stained nuclei. Scale bar, 30 µm. **b** Quantification of representative data in **a** from *n* = 4 independent experiments. Data for WT were set to 100%. One sample two-tailed Student’s *t*-test with a theoretical mean of 100, ^*^p = 0.0237, *t* = 4.264, df = 3. **c** PI(3)P levels in HeLa cells depleted of Rab35 and/or MTMR2 normalized to scrambled (scr) controls. See images in Supplementary Fig. 7c. Mean PI(3)P intensity (% of scr control) in CD63^+^ -compartments from n = 5 independent experiments. One-way ANOVA followed by Dunnett’s Multiple Comparison Test: Rab35 siRNA: *q* = 3.328, MTMR2 siRNA: *q* = 3.827, Rab35 siRNA + MTMR2 siRNA: *q* = 2.982, ^*^p = 0.01, df = 4. **d** Lysates from *Rab35* cKO astrocytes treated with DMSO, VPS34-IN1, SAR405, or Compound-19 (Cmp-19) were immunoblotted for Rab35, total S6K, p-S6K, and β-actin. **e** Quantification of representative data shown in d. p-S6K/S6K ratio (WT with DMSO set to 1). One sample two-tailed Student’s *t*-test with theoretical means of 1; *n* = number of independent experiments: DMSO: *n* = 6, ^**^p = 0.0053, *t* = 4.701, df = 5; 5 µM VPS34-IN1: *n* = 3, *p* = 0.1374, *t* = 2.411, df = 2; 10 µM VPS34-IN1: *n* = 6, *p* = 0.3368, *t* = 1.062, df = 5. n.s. non-significant. **f, g** Astrocytes from *Rab35*^*flox/flox*^ cKO or non-Cre^ER^-expressing *Rab35*^*flox/flox*^ control mice (WT) transduced with lentiviruses encoding scr or anti-MTMR2 shRNA were treated with DMSO or 10 µM VPS34-IN1 before analysis. **f** Representative immunoblots for p-S6K, S6K, eGFP (scr), MTMR2, Rab35, and β-actin. **g** Quantification of representative data shown in f from n = 5 independent experiments. p-S6K/S6K ratio (scr-WT astrocytes+DMSO set to 1). Paired one-tailed Student’s *t*-test; DMSO: ^*^p = 0.021, *t* = 2.948, df = 4; VPS34-IN1: *p* = 0.2114, *t* = 0.8920, df = 4. n.s. non-significant. Data represent mean ± SEM. Source Data file for numerical source data and unprocessed blots.

mTORC1 activation at late endosomes or lysosomes is potently stimulated by PI 3-phosphates such as the MTMR substrates PI(3)P and PI(3,5)P_2_. Hence, the observed elevation in mTORC1 signaling in the absence of Rab35 might conceivably be a consequence of increased PI 3-phosphate levels due to impaired hydrolysis mediated by MTMR2/MTMR13 and related MTMR complexes. While no reliable probes exist to quantitatively monitor the levels and distribution of PI(3,5)P_2_ in eukaryotic cells, PI(3)P can be semi-quantitatively measured by specific PI(3)P-binding probes such as recombinant eGFP2xFYVE. We used eGFP2xFYVE as a surrogate measure for the possible accumulation of MTMR substrates in Rab35 depleted cells. Loss of Rab35 in cKO astrocytes caused a significant, more than two-fold elevation in the levels of PI(3)P (Fig. 9a, b). Vps34 lipid kinase produces the bulk of PI(3)P in mammalian cells, which then serves as a precursor for PI(3,5)P_2_ synthesis by the PIKFYVE complex (comprised of the active PI 5-kinase PIKFYVE, the lipid phosphatase FIG4, and Vac14 protein). Consistently, we found that PI(3)P was strongly depleted following application of the selective Vps34-class III PI 3-kinase inhibitor VPS34-IN1 (Supplementary Fig. 7b). Similar observations were made in HeLa cells depleted of either Rab35, MTMR2, or of both proteins in combination, where PI(3)P was seen to accumulate on CD63-positive late endosomes or lysosomes (Fig. 9c, Supplementary Fig. 7), e.g. compartments where active mTORC1 is located. Next, we probed whether elevated PI 3-phosphate levels were causal for mTORC1 hyperactivity in *Rab35* cKO cells. Treatment of *Rab35* cKO astrocytes with either VPS34-IN1^49^, SAR405^50^, or Compound 19, selective inhibitors of Vps34-mediated PI(3)P synthesis, potently suppressed elevated mTORC1 signaling (Fig. 9d, e). Pharmacological inhibition of Vps34 activity also suppressed mTORC1 hyperactivation in *Rab35* cKO astrocytes co-depleted of MTMR2 (Fig. 9f, g). We conclude that loss of Rab35 and/or its downstream effector MTMR2 causes the accumulation of PI 3-phosphates including PI(3)P at late endosomes/lysosomes, resulting in local mTORC1 hyperactivation.

### Inhibition of mTORC1 or PIKFYVE rescues hypermyelination

**Fig. 10:**
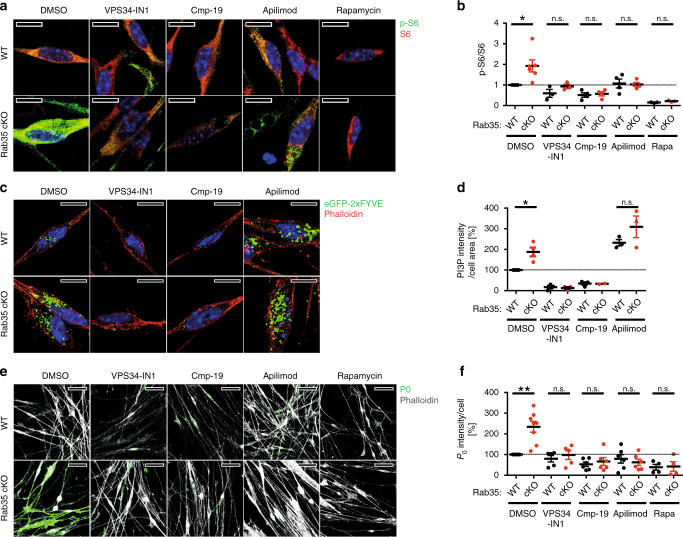
Inhibition of mTORC1 or PIKFYVE rescues hypermyelination in Rab35 KO cells. **a**, **b** Schwann cells from *Rab35*^*flox/flox*^ cKO or non-Cre^ER^-*Rab35*^*flox/flox*^ mice (WT) mice treated with DMSO, VPS34-IN1, Cmp-19, Apilimod, or Rapamycin. **a** Images of DMSO- or inhibitor-treated *Rab35* WT and cKO cells immunostained for p-S6 (green), total S6 (red). DAPI (blue), nuclei. **b** Quantification of representative data in a. Normalized (to DMSO-WT) p-S6/S6 ratio from n experiments. One sample two-tailed Student’s *t*-test (theoretical means of 1). DMSO: *n* = 6, ^*^p = 0.0249, *t* = 3.168, df = 5; VPS34-IN1: *n* = 3, *p* = 0.2059, *t* = 1.848, df = 2; Cmp-19: *n* = 4, *p* = 0.5464, *t* = 0.6779, df = 3; Apilimod: *n* = 4, *p* = 0.7586, *t* = 0.3366, df  = 3; Rapamycin: *n* = 3, *p* = 0.2241, *t* = 1.739, df = 2. **c** Images of differentiated DMSO- or inhibitor-treated *Rab35* WT or cKO Schwann cells. PI(3)P (green), F-actin (red), DAPI (blue), and nuclei. **d** Quantification of representative data in **c** from *n* experiments. Normalized (to DMSO-WT set to 100%) PI(3)P intensity/cell area. One sample two-tailed student’s t-test (theoretical mean of 100). DMSO: *n* = 4, ^*^p = 0.027, *t* = 4.058, df = 3; Apilimod: *n* = 3, *p* = 0.3237, *t* = 1.298, df = 2. **e** Images of inhibitor-treated *Rab35* WT or cKO Schwann cells. P0 (green), F-actin (gray). **f** Quantification of representative data in e from n experiments. Intensity of P0/cell number (in percentage, DMSO-WT as 100%) (see Supplementary Fig. 8i). One sample two-tailed student’s t-test (theoretical means of 100). DMSO: *n* = 8, ^**^p = 0.0014, *t* = 5.102, df = 7; VPS34-IN1: *n* = 5, *p* = 0.2093, *t* = 0.1.495, df = 4; Cmp-19: *n* = 6, *p* = 0.2510, *t* = 0.1.298, df = 5; Apilimod: *n* = 6, *p* = 0.3190, *t* = 0.7627, df = 5; Rapamycin: *n* = 4, *p* = 0.7541, *t* = 0. 3432, df = 3. Scale bars, 10 µm (**a**, **c**) or 50 µm (**e**). n.s. non-significant in all panels. Mean ± SEM. See Source Data file for numerical source data and unprocessed blots.

These combined data open the possibility that uncontrolled myelin growth due to lack of Rab35 might be rescued by pharmacological manipulation of PI 3-phosphate levels in Schwann cells. To test this, we finally analyzed the effects of pharmacological perturbation of PI 3-phosphate synthesis on mTORC1 activity and myelin protein expression in differentiated Schwann cells in culture. We probed mTORC1 signaling in primary cultures of Schwann cells from *Rab35*^*flox/flox*^ (“WT” control) or *Rab35*^*flox/flox*^ *CAG-Cre*^*ER*^ (cKO) treated with tamoxifen to acutely eliminate Rab35 expression. As expected *Rab35* cKO Schwann cells displayed significantly elevated p-S6/total S6 levels compared to WT controls and these were robustly suppressed by the mTORC1 inhibitor Rapamycin (Fig. 10a, b, Supplementary Fig. 8a). Increased mTORC1 activity in cKO Schwann cells was paralleled by increased levels of PI(3)P (Fig. 10c, d, Supplementary Fig. 8b).

If the elevated mTORC1 activity in *Rab35* cKO Schwann cells was a consequence of elevated PI(3)P levels, it should be rescued by pharmacological inhibition of Vps34-mediated synthesis of PI(3)P but not by selective inhibition of PI(3,5)P_2_ synthesis in the presence of the specific PIKFYVE inhibitor Apilimod. Rescue of elevated mTORC1 signaling in *Rab35* cKO Schwann cells by either Vps34 inhibitors or Apilimod, however, would indicate a major role for PI(3,5)P_2_ in mTORC1 hyperactivation in these cells. To test these hypotheses cultured differentiating Schwann cells were chronically treated with either DMSO (as a solvent control), low-doses of Rapamycin, the specific Vps34 inhibitors VPS34-IN1, or compound 19, or the selective PIKFYVE inhibitor Apilimod^51^. Elevated mTORC1 signaling in *Rab35* cKO Schwann cells was potently rescued by pharmacological inhibition of Vps34-mediated synthesis of PI(3)P or inhibition of the PI(3,5)P_2_ synthesizing enzyme PIKFYVE (Fig. 10a, b; Supplementary Fig. 8a). Semi-quantitative determination of PI(3)P levels in Schwann cell cultures confirmed the near complete elimination of PI(3)P production by Vps34 inhibition, while PI(3)P levels were elevated under conditions of impaired synthesis of PI(3,5)P_2_ from PI(3)P in the presence of Apilimod (Fig. 10c, d; Supplementary Fig. 8b). The levels of PI(4)P analyzed as a control remained unchanged (Supplementary Fig. 8c, d). Elevated mTORC1 activity was also observed in WT and *Rab35* cKO Schwann cells lentivirally depleted of MTMR2 (Supplementary Fig. 8e–h). These findings suggest that elevated mTORC1 activity in the absence of Rab35 is largely caused by the accumulation of PI(3,5)P_2_ in Schwann cells, possibly as a consequence of perturbed PI(3,5)P_2_ hydrolysis by MTMRs including MTMR2/MTMR13 consistent with previous data^41^, although more indirect mechanisms (e.g. via recycling) cannot be ruled out. Finally, we assessed the effects of pharmacological manipulation of PI 3-phosphate synthesis on myelin protein production by *Rab35*^*flox/flox*^ WT control or cKO Schwann cells in culture. Acute tamoxifen-induced cKO of Rab35 in cultured differentiated Schwann cells from *Rab35*^*flox/flox*^ mice resulted in increased production of myelin protein zero (P0) compared to WT controls and this defect was rescued by inhibition of mTORC1 signaling in the presence of Rapamycin. Strikingly, we found that increased myelin protein P0 synthesis was equally well suppressed by sustained pharmacological inhibition of either PI(3)P or PI(3,5)P_2_ synthesis by Apilimod (Fig. 10e, f; Supplementary Fig. 8i).

These collective data indicate that uncontrolled myelin growth in Rab35-depleted Schwann cells largely results from mTORC1 hyperactivation caused by the accumulation of the MTMR substrate PI(3,5)P_2_, and possibly also PI(3)P, at late endosomes or lysosomes. They further suggest that pharmacological manipulation of mTORC1 signaling and/or of PI 3-phosphate metabolism may represent a potential avenue for the treatment of CMT4B1/2-type hereditary neuropathies caused by loss of myotubularin family phosphatases.

## Discussion

Previous work had revealed critical roles for the myotubularin-family PI 3-phosphatases MTMR2 and MTMR13, and for the AKT-mTORC1 signaling pathway in the control of myelin growth and architecture. In our study we identify the small Ras-family GTPase Rab35 as a regulator of myelination that by association with MTMR13 and MTMR2 downregulates lipid-mediated mTORC1 activation and thereby restricts myelin growth. Several lines of evidence support this conclusion: (i) Rab35•GTP physically and functionally associates with MTMR13-based myotubularin complexes in vitro and in living cells. (ii) Genetically encoded loss or depletion of Rab35 phenocopies MTMR2 depletion with respect to the accumulation of PI 3-phosphates, e.g. substrates of MTMR activity, and elevated mTORC1 activity in a variety of cell models including primary astrocytes and differentiated Schwann cells in culture derived from tamoxifen-inducible *Rab35*^*flox/flox*^ cKO mice. (iii) Schwann cell-specific cKO of Rab35 in mice causes focal hypermyelination and mTORC1 hyperactivity in the peripheral nervous system. These alterations are cell autonomous defects of myelinating Schwann cells and phenotypically resemble MTMR2 loss-of-function or conditions of elevated mTORC1 activity, e.g. induced by conditional loss of PTEN, suggesting that Rab35 acts, at least in part, by MTMR-mediated regulation of mTORC1 activity to control myelin growth. (iv) Finally, we demonstrate that focal hypermyelination defects in *Rab35* cKO mice or Schwann cells are ameliorated by pharmacological inhibition of mTORC1 activity in vivo and in vitro or by blockade of PI(3,5)P_2_ phosphate synthesis by Apilimod or upstream inhibition of Vps34 kinase-mediated production of PI(3)P.

These combined findings are most consistent with a model in which focal hypermyelination in the absence of Rab35 in Schwann cells arises from the defective function of MTMR phosphatase complexes, resulting in the accumulation of PI(3)P and, likely, PI(3,5)P_2_ (although current methodology does not allow us to directly assess this in Schwann cells), which facilitate mTORC1 activation (consistent with^10–12^). Such a model agrees well with recent findings that suggest defective PI 3-phosphate metabolism or recognition, e.g. due to mutations in PTEN, MTMR2, MTMR13, or in the FYVE domain (i.e. a module that binds PI 3-phosphates)-containing protein Frabin/FGD4, as a common feature of neuropathies with myelin overgrowth^3,4,14,43^. Our findings are further consistent with a critical dual function for mTORC1 signaling - a process crucially regulated by PI 3-phosphate metabolizing enzymes such as PTEN - in controling the differentiation of myelin producing cells and myelin growth^14,16,46^. How exactly the different forms of overgrown myelin such as myelin outfoldings and tomacula arise, remains poorly understood. Akin to results for PTEN mutants^14^ we found that mTORC1 inhibition ameliorated tomacula but much less well myelin outfoldings in *Rab35* cKO^SC^ mice, suggesting that mTORC1 activity primarily controls bulk membrane addition. It is therefore conceivable that other pathways beyond mTORC1 signaling contribute to focal hypermyelination in *Rab35* cKO^SC^ mice. These may include dysregulation of exosome secretion, a process known to be important for myelin membrane growth that is regulated by the Rab35 GTPase activating proteins TBC1D10A-C^23^, phosphoinositide regulation of actin dynamics by Rab35^21,52^ and/or MTMRs, or the endocytic recycling of signaling receptors, for example as a consequence of alterations in the activity of the small GTPase Arf6^21,53^ via the Rab35 effector ACAP2^20,21,28,54^. The latter possibility is consistent with the finding that depletion of Rab35 or ACAP2 promotes myelin protein synthesis in oligodendrocytes in culture^32^, while Arf6 itself has been implicated in Schwann cell differentiation^55^.

Unlike its interaction partners MTMR13 or MTMR2 (bound to Rab35 via MTMR13 or MTMR5) Rab35 does not appear to be an inherited neuropathy disease gene. This is likely owed to the fact that Rab35 function is essential for survival, at least in mice, and that mutations within Rab35 are unlikely to affect a single effector protein and, hence, would exhibit pleiotropic effects in many different cell types and organs. Given the phenotypic similarity of *Rab35* cKO^SC^ in Schwann cells to focal hypermyelination observed in MTMR2 and MTMR13 mouse models^42,45,56^ and the physical and functional interaction of Rab35•GTP with these proteins, we propose that Rab35 is a major upstream regulator of MTMR function in Schwann cells and other cell types. The fact that Rab35•GTP can bind to both MTMR13 and MTMR5 and either of these catalytically inactive proteins is capable of associating with several active MTMRs^57^ including MTMR2 and MTMR1(compare Fig. 1, Supplementary Fig. 1) suggests that Rab35 acts as a master regulator for MTMR-mediated hydrolysis of PI 3-phosphates at late endosomes or lysosomes. Consistent with this hypothesis we observe that focal hypermyelination in peripheral nerves from *Rab35* cKO^SC^ mice is even more severe than in *Mtmr2* or *Mtmr13* KO models^42,45,56,58^. Rab35-bound MTMRs (e.g. including MTMR13, MTMR5 and their associated active MTMs such as MTMR2, MTMR1, and possibly others) in this model would counteract the role of the PI(3,5)P_2_-metabolizing PIKFYVE-Vac14-FIG4 complex, a genetic interactor of *Mtmr2*^7^. Indeed, PIKFYVE-Vac14-FIG4 are required for oligodendrocyte differentiation and myelination in the central nervous system^7,59^.

In conclusion, our findings suggest an intimate relationship between the synthesis and turnover of PI 3-phosphates mediated by Rab35-associated MTMRs and the activity of late endosomal/lysosomal mTORC1 that crucially controls myelin growth, thereby unraveling a common effector pathway for myelination in the peripheral nervous system. Based on this model it appears that interference with the synthesis of PI 3-phosphates, in particular of PI(3,5)P_2_ and/or pharmacological perturbation of mTORC1 signaling may represent viable options for the treatment of CMT patients suffering from inherited neuropathies with abnormal myelin growth, e.g. by local administration of mTORC1 or PIKFYVE inhibitors.

## Methods

### Antibodies

Primary and secondary antibodies and probes used for immunoblotting and immunocytochemistry are specified in Supplementary Data 2.

### Primers

Primers used for analytical (genotyping) and preparative (cloning) PCR-amplification are listed in Supplementary Data 2.

### shRNAs

pLKO.1 encoded shRNAs, non-targeting scrambled with parallel GFP-expression (RHS6848), mouse Mtmr2 targeting shRNA (TRCN0000030098), and mouse targeting Rab35 (TRCN0000100532)

were obtained from Dharmacon.

Mtmr2 shRNA: 5‘-AAAGGACATGATTGGAGTAGC-3; Rab35 shRNA: TTGTCTGCGAATCGTAACAGC; scrambled shRNA: 5’-ACCGGACACTCGAGCACTTTTTGAATTC-3’.

### siRNAs

Used siRNAs were 21-mers including 3′dTdT overhangs: MISSION® siRNA Universal Negative Control #1 (Sigma); huRab35: 5′-AGAAGAUGCCUACAAAUUU-3′; huMTMR2: 5′-GAAAAUGGGUGGAAGCUAU-3′. huMTMR13: Sbf2 ON-TARGETplus siRNA, J-014684-09 (Dharmacon); huMTMR5: Sbf1 ON-TARGETplus siRNA J-021405-05 (Dharmacon); huMTMR13 SmartPool: ON-TARGETplus Human SBF2 siRNA smart pool, L-014684-01 (Dharmacon); huMTMR5 SmartPool: ON-TARGETplus Human SBF1 siRNA smart pool, L-021405-00 (Dharmacon).

### Inhibitors

All inhibitors were dissolved in DMSO to stock solutions as indicated in Supplementary Table 2. Working concentrations are indicated in the method section for each experiment.

### Animals

*Rab35*^*flox/flox*^ were generated by homologous recombination using mouse genomic DNA harboring the *Rab35* locus isolated from the BAC clone RP23-192F6 (http://bacpac.chori.org/). *LoxP* recombination sites and a neomycin selection cassette were introduced flanking the second and third coding exons of the mouse Rab35 genomic locus. The resulting targeting vector was linearized by *Not*I digestion, and ES cells (F1 from 129Sv/C57BL6J) were electroporated. After positive and negative selection with Geneticin and ganciclovir, respectively, genomic DNA of surviving ES cell colonies were screened by nested long-range PCR using primers outside the 5′ (Scr-F1, Scr-F2, PL452-LoxP-Sc1R and PL452_Sc5R2) and 3′(PL452_3′-Sc1F, PL451_3′-Sc2F, Scr-R1 and Scr-R2) arms. Targeted ES clones were expanded and confirmed by long-range PCR. Correctly targeted ES cell clones were used to generate chimeric mice at the NCI gene targeting facility. Chimeric mice were bred with β-actin–flippase (flp) transgenic mice (The Jackson Laboratory) to remove the PGKneo cassette, resulting in heterozygously floxed *Rab35*^*flox/+*^ mice that were confirmed by PCR products using LoxP5F, PL452-LoxP-Sc1R, and Frt-3R primer and the lack of a product from NeoF and NeoR primers which recognize the PGKneo cassette. Litters were intercrossed to obtain homozygotes (*Rab35*^*flox/flox*^). The mice were housed in a specific pathogen-free facility and cared for in accordance with National Institutes of Health guidelines, and all protocols were approved by the National Cancer Institute Animal Care and Use Committee.

*Rab35*^*flox/flox*^ animals were either crossed with a tamoxifen inducible *Cre*^*ER*^ mouse line (B6.Cg-Tg (*CAG-Cre*/Esr1*)5Amc/J; The Jackson Laboratory) for in vitro recombination or a *P0-Cre* line (ref. ^44^) for in vivo recombination. The presence of *Rab35* wild-type allele in the heterozygotes of the F1 generation was detected with ‘Lox5F’ as forward primer and ‘Lox5R’ as reverse primer resulting in an amplification of 573 bp from the intron 1 region. Replacing the reverse primer by ‘PL452-Loxp-Sc1R’ allowed for detection of the inserted *loxP* site (647 bp) in the mutant floxed allele. *CAG-Cre*^*ER*^ transgene was detected by PCR-amplification of 445  bp using ‘TM63_EllaCre_fw’ and ‘TM64_EllaCre_rev’ primer. For *P0-Cre* detection the following primers were used: P0_fw and P0_rev. For PCR, we isolated DNA from tail biopsies using DirectPCR lysis reagent (Viagen Biotech), following manufacturer’s directions. *Mtmr2*^−/−^ mice have been already reported and characterized^45,56^.

All animal experiments were conducted according to the guidelines of the “Landesamt für Gesundheit und Soziales” (LAGeSo) and with their permission under the license S0313/17 and T0243/08, the French national regulations according to guidelines 2010/63/UE, and the Italian national regulations and covered by experimental protocols reviewed by local Institutional Animal Care and Use Committees (IACUC #894).

### In vivo analyses

Animals were randomly included into experimental groups according to genotyping, age and sex. *Rab35* cKO^SC^ are mice with conditional ablation of *Rab35* in Schwann cells (*Rab35*^*flox/flox*^ *P0-Cre*). These mutants were compared with controls, which correspond to *Rab35*^*flox/flox*^ or *Rab35*^*flox/+*^ mice which are phenotypically normal. No animals had to be excluded due to illness in all the experiment performed. Investigators performing animal handling, sampling, euthanasia, and raw data analysis were not blinded.

Semithin section and ultrastructural analyses of sciatic nerves were performed as follows:^45,56^ Tissues were removed and fixed with 2% glutaraldehyde (vol/vol) in 0.12 M phosphate buffer, postfixed with 1% osmium tetroxide (vol/vol), and embedded in Epon (Fluka). Semi-thin sections (0.5–1 μm thick) were stained with toluidine blue and examined by light microscopy. Ultrathin sections (70–90 nm thick) were stained with uranile acetate and lead citrate and examined by electron microscopy.To perform morphometric analysis, digitalized images cross sections were obtained from corresponding levels of sciatic nerves with a ×100 objective and Leica DFC300F digital camera (Milan, Italy). At least five images per animal were analyzed using the Leica QWin software (Leica Microsystem) to calculate the g-ratio that is the ratio between the mean diameter of an axon (without myelin) and the mean diameter of the same axon including the myelin sheath. To estimate the percentage of myelinated fibers carrying alterations the entire nerve section was reconstructed and the total number of myelinated fibers was assessed. For morphometric analysis on ultrastructural sections, 20–40 images per animal were taken using TALOS L120C transmission electron microscope (Thermo Fisher Scientific,Waltham, MA, USA) at 120 kV using 3400×(for P20) and the g-ratio values were determined by measuring axon and fiber diameters.

Sciatic nerve lysates from *Rab35* cKO^SC^ and controls for western blot analysis were prepared using a lysis buffer containing 2% SDS, 50 mM Tris buffer pH 8.0, 150 mM NaCl, 10 mM NaF, 1 mM NaVO_3_, complete protease and phosphatase inhibitors (Roche). Protein quantification was performed using BCA assay (Pierce, Thermo Fisher Scientific). Rapamycin (LC Laboratories) was dissolved in Ethanol and administered QD by i.p. injection 5 days a week at a final concentration of 10 µg/g in vehicle solution containing 5% Polyethylen glycol 400 (PEG 400), 5% TWEEN 80. and NaCl 0.9%, as already reported^14^.

### Cell lines

GFP-Rab35^endo^ HeLa cells were derived from a TALEN-edited HeLa knock in (KI) cell line, which expresses endogeneous Rab35 tagged with GFP^30^. HEK293T and HeLa cells were obtained from ATCC. Cells were cultured in DMEM with 4.5 g/L glucose (Thermo Fisher) containing 10% heat-inactivated FBS (Gibco) and 100 U/mL penicillin and 100 µg/mL streptomycin (Gibco) during experimental procedures and were routinely tested for mycoplasma contamination.

### Preparation of primary astrocytic cultures

Primary astrocytic cultures were prepared by isolating cortices from tamoxifen-inducible cKO *Rab35* mice (*Rab35*^*flox/flox*^ *CAG-Cre*^*ER*^*)* for knockout cultures and from littermates without Cre-expression for WT cultures at postnatal days P2 to P5 as follows. Cerebral cortices were isolated and meninges were removed in ice-cold HBSS (Thermo Fisher). The tissue was digested for 15 min at 37 °C in TrypLE (Thermo Fisher) supplemented with 150 U/mL DNase I (Sigma). Cells were dissociated by trituration in DMEM with 4.5 g/l glucose (Thermo Fisher) supplemented with 10% (v/v) fetal bovine serum (FBS) and 100 U/mL penicillin and 100 µg/mL streptomycin (‘culturing medium’) with 375 U/mL DNase I. Cells were pelleted by centrifugation, filtered through a 70-µM Nylon cell strainer (Sigma) and plated in culturing medium in 10 cm dishes. At day in vitro (DIV) 1, cells were washed with D-PBS (Thermo Fisher) and WT and cKO cultures were supplied with fresh culturing medium supplemented with 0.4 µM tamoxifen ((Z)-4-hydroxytamoxifen, Sigma) for knockout induction. Culturing medium supplemented with tamoxifen was exchanged every 2–3 days. Cells were passaged at DIV8 to well plates or PLL-coated coverslips at low densities to reach confluency at the day of experiment, performed at DIV20 to DIV22. For protein quantification under serum-deprived conditions, the day before lysis cells were rinsed once with D-PBS and supplied with culturing medium without FBS (‘-FBS’) over night. For protein quantification upon acute inhibitor treatment, astrocytic cultures were supplied with DMSO, 5 or 10 µM VPS34-IN1, 30 µM SAR405 or 30 µM Cmp-19 1 h before cell lysis.

### Preparation of primary Schwann cell cultures

Schwann cells were purified from sciatic nerve and Brachius plexus, isolated from P4 to P6 mice after decapitation. Nerves were collected in Leibovitz’s L-15 medium (Thermo Fisher) and remaining connective tissue and endoneurium were removed prior to digestion in L-15 supplemented with 0.1 % (w/v) Collagenase A (Sigma) and 0.25 % (w/v) Trypsin (Thermo Fisher) for 1 hr at 37 °C and 5% CO_2_. After dissociation by trituration, nerve tissue was pelleted in DMEM with 4.5 g/L glucose, GlutaMAX and pyruvate (Thermo Fisher) supplemented with 5% (v/v) heat-inactivated horse serum (Thermo Fisher) and 100 U/mL penicillin and 100 µg/mL streptomycin (Gibco) (‘plating medium’). One 6-well for each four dissociated nerves was coated for 3 h at room temperature (RT) with 20 µg/mL PLL (Sigma) in water and subsequently for 1 h at 37 °C with 10 µg/mL Laminin (Sigma) in DMEM, before Schwann cells were added in plating medium supplemented with 5 µM cytosine β-d-arabinofuranoside (AraC, Sigma).

At DIV3 Schwann cells were passaged to 20 µg/mL PLL and 20 µg/mL Laminin coated 12 mm coverslips with a density of 15.000 cells/15 µL. The cells were cultured in supplemented defined DMEM-SATO medium: DMEM with 4.5 g/L glucose (Thermo Fisher), 2 mM l-glutamine, 5 µg/mL insulin, 5 µg/mL *N*-Acetyl-cysteine, 10 ng/mL d-Biotin (Sigma), 1x trace elements B (Thermo Fisher), 1x B27-supplement (Thermo Fisher), 1.5 mM BSA, 1.3 mM apo-transferrin bovine, 0.1 mM putrescine, 0.2 µM progesterone, 0.23 µM sodium selenite, and 40 pg thyroid hormone triiodothyronine (all from Sigma). In all, 50 µg/mL ascorbic acid, 1 µM Forskolin and 20 µg/mL Neuregulin-1/Heregulin ß-1 (all from Sigma) for differentiation, and myelination induction and 0.4 µM tamoxifen ((Z)-4-hydroxytamoxifen, Sigma) for knockout induction were applied to WT and cKO cultures from DIV3-4 on. Half of the medium was exchanged every two to three days. DMSO or inhibitors for chronical application in rescue experiments (p-S6/S6- or myelin protein P0- immunostainings) were added to the medium from DIV6-7 on: 1 µM VPS34-IN1, 2.5 µM Cmp-19, 50 nM Apilimod or 15 nM Rapamycin. For acute inhibition with VPS34 inhibitors (PI(3)P immunolabelling) 10 µM VPS34-IN1 or 10 µM Cmp-19 were applied for 1 h prior to fixation. Cells were fixed for immunocytochemistry on DIV11 to DIV12.

### Preparation of Schwann cell/DRG organotypic explants

Myelin-forming Schwann cell/DRG neuron co-cultures were established from E13.5 mouse embryos generated from *Rab35*^*flox/flox*^ mice crossed with *Rab35*^*flox/flox*^ *P0-Cre*. To obtain efficient *P0-Cre* mediated recombination of the *Rab35*^*flox*^ locus ex vivo, organotypic explants were kept in Neurobasal (NB) medium supplemented with 2.5 S NGF (Nerve growth factor) at 50 ng/ml final concentration and B27 (Thermo Fisher) for 8–9 days prior to induce myelination. For myelination, C-media supplemented with ascorbic acid for additional 15 days (50 μg/ml, SIGMA) was used^60^. To perform immunohistochemistry, co-culture explants were fixed for 15 min in 4% paraformaldehyde, permeabilized for 5 min in ice-cold methanol at -20°C, blocked for 20 min with 10% NGS, 1% BSA and then incubated with primary antibodies for 1 hour at room temperature in PBS 1×(anti-Mbp) and overnight in 5%BSA, 1% NGS, 0.6% Triton in PBS1x at 4°C (anti-NF-M). After washing, the coverslips were incubated with the secondary antibody for 30 min, washed and mounted. Transduction of Schwann cell/DRG neuron co-culture explants was performed as already reported^61^. To quantify the amount of myelinated segments, using a fluorescence microscope at least 5–10 fields/coverslip were randomly acquired and Mbp-positive myelinated fibers were counted per field. Means of each coverslip/DRG have been used as different “*n*” for statistical analysis. To quantify myelinated fibers carrying abnormalities, at least 300 Mbp-positive myelinated fibers were evaluated, from “*n*” different DRG explants/coverslips using a TCS SP5 laser-scanning confocal microscope (Leica). The percentage of Mbp-positive fibers showing altered myelin among the total number of Mbp-positive fibers was indicated. Two independent experiments were performed.

### Plasmids

A complete list of all DNA-plasmids used in this study is provided in Supplementary Data 2. N-terminally FLAG-tagged MTMR13 in a pcDNA3.1 backbone was a kind gift from Gilbert di Paoli and was transferred into a pcDNA3.1-HA backbone, removing the HA-tag, using 5′-KpnI and 3′-EcoRV restriction sites. For the construction of mCherry-MTMR13, FLAG-tag was exchanged by mCherry from pmCherryC1 using restriction sites 5′-KpnI and 3′-EcoRV For mCherry-tagged MTMR13 domains, full-length MTMR13 (FL; aa1-1848; 5547 bp) was used as a PCR template. The DENN-domain truncation mutant of MTMR13 (MTMR13ΔDENN) and the various MTMR13 domains were amplified using primers specified in the primer table (Supplementary Data 2): MTMR13ΔDENN (aa493-1849): 4071 bp; DENN domain (aa1-471): 1413 bp; PH-GRAM domain (aa806-1018): 642 bp; phosphatase (PTP) domain (aa1100-1591): 1479 bp; PTP + coiled coil (CC) region (aa1100-1591): 1797 bp; PH domain (aa1743-1849): 324 bp. The respective PCR products were inserted into a pcDNA3.1-based mCherry-containing vector using restriction sites 5′- NotI and 3′-XbaI for MTMR13ΔDENN, 5′-EcoRV and 3′-NotI for DENN and PH-GRAM domains. For PTP, PTP + CC region and PH domains, BamHI was used at the 5′-position. Using pUAST- YFP-Rab35mouse (gift from Matthew Scott, Addgene plasmid # 46014) as a PCR template, GST-Rab35 was subcloned by insertion of amplified Rab35 into pGEX4T-1 using 5′-EcoRI and 3′-NotI restriction sites. 540 bp from the 5′-end of Rab35 were amplified and inserted into pGEX4T-1 using the same restriction sites to generate GST-Rab35ΔC (aa1-180). Rab35 was inserted into pcDNA3.1_mycBioID (BirA*), a gift from Dr. Kyle Roux (Sanford Research/University of South Dakota, Sioux Falls, SD, USA), with restriction sites 5′-EcoRV and 3′-HindIII to obtain pcDNA3.1_BirA*-Rab35. pcDNA3.1_eGFP-Rab35 was generated by insertion of amplified Rab35 C-terminal to eGFP using restriction sites 5′-XhoI and 3′-EcoRI. pcDNA3.1_eGFP-Rab35CA(Q67L) and_eGFP-Rab35DN(S22N) were generated by QuickChange site-directed mutagenesis (Agilent). pEGFPC2_mCherry-MTMR5 was obtained by exchanging the N-terminal eGFP-tag for mCherry in pEGFPC2_GFP-MTMR5 (gift from Michael Clague) at restriction sites 5′-AgeI and 3′-HindIII. GST-Rab1A, -Rab5, -Rab7, and -Rab11 were a gift from Dr. Mitsunori Fukuda (Tohoku University, Sendai, Japan). pCMV6_myc-MTMR2ms (NM_023858) was obtained from origene (#MR215223). pcDNA3.1_eGFP-MTMR2ms was subcloned by insertion of amplified MTMR2ms C-terminal to eGFP using restriction sites 5′-XhoI and 3′-ApaI. pcDNA3.1_eGFP-MTMR2ms-C417S was generated by QuickChange site-directed mutagenesis (Agilent). N-terminally eGFP-tagged MTMR2hu, MTM1 and MTMR1 in pcDNA3.1 based vectors are described elsewhere^62^.

### Cell transfection

HeLa cells were transfected with plasmid-DNA using JetPRIME® (Polyplus). For siRNA-mediated knockdown in HEK293T or HeLa cells, reverse transfection using the same reagent was performed and cells were harvested after 96 h. For Large-scale transfection with plasmids for transient overexpression of proteins in HEK293T or

eGFP-Rab35^endo^ KI HeLa cells for biochemical analysis, calcium phosphate transfection was used. For rescue experiments upon siRNA-mediated protein depletion, HEK293T cells were transfected with plasmid-DNA 48 h after reverse transfection with siRNA and harvested after transient overexpression for 48 h.

### Preparation of lysates from cell cultures

Mammalian cell cultures were rinsed once with ice-cold PBS before cell lysis buffer (20 mM HEPES pH 7.4, 100 mM KCl, 2 mM MgCl_2_, 1 % (v/v) Triton X-100) freshly supplemented with 1 mM PMSF, 0.3% protease inhibitor cocktail (Sigma) and phosphatase inhibitors (coctails 2 and 3, Sigma) was applied. Cells were collected and incubated for 20 min on ice followed by centrifugation for 5 min at 17,000×*g*. Lysates used for affinity chromatography or immunoprecipitation were further cleared by ultracentrifugation at 180,000 × *g* for 15 min at 4 °C in a TLA110 rotor (Sorvall).

Protein concentration was determined using Bradford assay. Upon addition of Laemmli-sample buffer (boiled 5 min at 95 °C, pelleted 5 min at 17,000 × *g*), samples containing 10 µg to 40 µg protein amount were resolved by SDS-PAGE and analysed by subsequent immunoblotting, using HRP-coupled or LI-COR infrared-fluorescent secondary antibodies.

### Immunocytochemistry of cell cultures

Cell cultures grown on coverslips were rinsed with 1x phosphate buffered saline (PBS) and fixed with 4% (w/v) paraformaldehyde (PFA) with 4 % (w/v) sucrose for 15 min at room temperature (RT). Cells were simultaneously permeabilized and blocked for 1 h in PBS supplemented with 0.3% (v/v) Triton X-100 and 10 % (v/v) normal goat serum (NGS). Primary and secondary antibodies were applied in blocking solution for 1 h at RT with PBS washes in between. Coverslips were mounted using Immu-Mount (Thermo Fisher) supplemented with 1 mg/mL DAPI (Sigma) to label cell nuclei. LAMP-2 was detected using a modified protocol: Triton X-100 was omitted. Permeabilization was performed with 20 µM digitonin in PBS for 5 min after fixation. PI(4)P and PI(3)P immunodetection was done as described^62^, for the latter using a reduced concentration of 0.05 µg/mL recombinantly expressed and purified eGFP-2xFYVE(Hrs) or a GST-tagged phox domain of P40 (‘phoxP40’); a kind gift from Dr. I. Ganley, MRC Protein Phosphorylation and Ubiquitylation Unit, College of Life Sciences University of Dundee, Dundee, UK. In brief, coverslips were rinsed with 1x PBS and fixed with 2 % (w/v) paraformaldehyde (PFA) with 2% (w/v) sucrose for 15 min at room temperature (RT). Cells were washed 3x with PBS, permeabilized for 5 min in a PIPES-based buffer (PIB; 20 mM PIPES, 137 mM NaCl, 2.7 mM KCl, pH 6.8) for 5 min at RT with 20 µM Digitonin, followed by three thorough washes with PIB. The cells were simultaneously blocked and labeled for 45 min with eGFP-2xFYVE (Hrs) or phoxP40 in PIB supplemented with 5% (v/v) NGS and 50 mM ammonium chloride. After washing in PIB, primary and secondary antibodies were applied in 5% (v/v) NGS in PIB for 1 h and 45 min, respectively. The coverslips were post-fixed for 5 min with 2% PFA fixative, and washed with PBS, three times with 50 mM ammonium chloride, and once without. Mounting was performed as described above.

### Confocal microscopy

Images of immunostained cell cultures were acquired on a Zeiss Laser Scanning confocal microscope (LSM) 710, routinely using a ×63 oil immersion (1.4 NA) DIC objective, and analyzed using ImageJ software, by normalizing sum fluorescence intensities of protein signals over Phalloidin positive cell area. P0-protein immunostaining in Schwann cell mono-cultures was imaged with a 10x dry (0.3 NA) objective. Sum intensities per image (10 images per condition and experiment) were normalized by the number of DAPI-positive cell nuclei per image. Whole cell images of HeLa cells or astrocytes immunolabeled for lipids were acquired with a z-stack series of usually seven slices with 0.5 µm intervals and summed up using the ImageJ ‘z-projection’ tool. For PI(3)P level analysis, a threshold was set to extract unspecific fluorescent signals.

### Affinity chromatography with GST-coupled Rab proteins

GST-Rab fusion proteins were expressed in a *E. coli* Rosetta™ (DE3) (Novagen) under optimized expression conditions with 100 µM Isopropyl-β-d-thiogalactopyranosid (IPTG) at 20 °C for 20 h. Bacteria pellets were either stored in PBS at −20 °C or directly processed for protein purification. Nucleotide-free purification of GST-coupled Rab proteins was performed in PBS supplemented with 2 mM EDTA, 0.02% (v/v) Cyanase, 0.5 mg/mL Lysozyme, 1 mM PMSF, 1 tablet/50 mL of EDTA-free Protease Inhibitor Cocktail (Sigma), and 150 mM NaCl. Cells were disrupted by sonification with subsequent addition of 1% (v/v) Triton X-100, followed by 15 min rotation at 4 °C. Bacterial lysates were cleared from cell debris by centrifugation at 35,000 × *g* for 15 min in a SS-34 rotor (Sorvall) at 4 °C, and subjected to affinity purification. GST-bind resin (Novagen) was used according to manufacturer’s instructions. In brief, 300 µl beads-slurry were washed with PBS and incubated with cleared bacterial lysates for 2 h at 4 °C while rotating. Beads with bound GST-fusion proteins were stored for 20 h at maximum in a phosphate-free buffer (20 mM HEPES pH 7.5, 5 mM EDTA).

10 cm culture dishes of HEK293T cells were lysed 24 h post-transfection and cleared by ultracentrifugation as described above.  For each sample, 100 µg of bead-coupled GST-fusion protein were washed three times in ice-cold 500 µl cell lysis buffer (without MgCl_2_) and supplemented with 5 mM EDTA. GST-Rab proteins were loaded with either 1 mM GTPyS or GDP (Sigma) in 50 µl cell lysis buffer (without MgCl_2_) at 30 °C for 15 min with slight agitation. Control beads with GST alone were supplemented with 5 mM EDTA instead of nucleotides. In all, 10 mM MgCl_2_ was added, followed by an incubation of 10 min at 4 °C. Lysates were freshly supplemented with PMSF and 1 mM GTPyS, GDP, or EDTA, and applied to the beads. After 2 h rotation at 4 °C, bead-coupled proteins were washed three times for 10 min using lysis buffer with reduced concentrations of 50 mM KCl and 0.5% (v/v) Triton X-100 and supplemented with 0.1 mM nucleotides, and a fourth time without detergent. Proteins were eluted in Laemmli-sample buffer at 95 °C for 5 min, and analyzed by SDS-PAGE and immunoblotting.

### Immunoprecipitation assays

Immunoprecipitation of eGFP-Rab35 from 15 cm culture dishes of eGFP-Rab35^endo^ KI HeLa cells, or overexpressed eGFP-Rab35CA or -DN from 10 cm culture dishes of HEK293T cells 24 h after transfection was performed using eGFP-Trap^®^_MA beads (Chromotek). Cleared lysates were applied to 20 µl lysis buffer-washed eGFP-Trap^®^_MA beads and incubated for 90 min rotating at 4 °C. Protein-coupled beads were washed three times with lysis buffer, with reduced concentrations of 50 mM KCl and 0.5% (v/v) Triton X-100, and once without detergent. Proteins were eluted in Laemmli-sample buffer for 5 min at 95 °C, analyzed by SDS-PAGE and immunoblotting.

### Lentiviral production in HEK293T cells

The production of lentiviral particles containing shRNA-encoding plasmids (pLKO.1 backbone, Dharmacon), using a 2nd generation lentiviral system according to the manufacturer’s guidelines (Dharmacon), and transduction of mammalian cells was done in accordance with the S2 guidelines (LAGESO). In brief, 10 cm dishes of HEK293T cells were co-transfected over night with 15 µg pLKO.1_GFP_shRNA-scrambled or pLKO.1_shRNA-msMTMR2, 10.5 µg lentiviral envelope VSV-G encoding plasmid pMD2.G and 4.5 µg lentiviral packaging-encoding plasmid psPAX2 using calcium-phosphate transfection. Cells were washed with D-PBS (Thermo Fisher) and fresh cell culture medium was applied. Viral particles were harvested by collecting the culturing medium after 24 h and 48 h with subsequent centrifugation at 0.2 × *g* for 5 min at room temperature. The virus-containing supernatant was filtered using a 0.45-µm falcon filter and viral particles were ×100 concentrated by centrifugation in Amicon Ultra-15 100 kDa filter columns (Merck) at 5000 × *g* at 4 °C, aliquoted and stored at −80 °C. The number of transducing particles were calculated by the number of GFP-positive HEK293T cells 72 h post-transduction. Titer concentration was usually between 10^8^ and 10^9^ Units/mL.

### Viral transduction of primary cultures

Viral transduction of astrocytic cultures was performed with a multiplicity of infection (MOI) of 20. Cells were transduced in two consecutive rounds, at DIV11-12 and DIV16-17, before harvest at DIV20-22. Primary Schwann cells in monocultures were transduced with a MOI of 50 in two consecutive rounds, at DIV3-4 and DIV7-8, and fixed on DIV11-12.

### BioID from HEK293T cells

In-cell proximity-dependent biotinylation (BioID) was performed according to^39^. In brief, 6 cm dishes of HEK293T cells were transfected with a construct encoding for myc-tagged promiscuous biotin protein ligase BirA* coupled N-terminally to Rab35 or BirA* alone as a control, and supplemented with cell culture medium containing 50 µM Biotin (Sigma, B4639) for 24 h. Lysates were prepared as described and added to Streptavidin-coupled agarose beads (Millipore) for affinity purification overnight. Bound biotinylated proteins were washed six times each 8 min rotating with four different buffers (2×: 2% SDS in H_2_O; 1×: 0.1% sodium deoxycholate, 1% (v/v) Triton X-100, 500 mM NaCl, 1 mM EDTA, 50 mM Hepes, pH 6.8; 1×: 250 mM LiCl, 0.5% NP-40, 0.5% sodium deoxycholate, 1 mM EDTA, 10 mM TRIS, pH 8.1; 2×: 50 mM NaCl, 50 mM TRIS, pH 7.4) and eluted from agarose-coupled streptavidin with Laemmli-sample buffer supplemented with an excess of 30 mM Biotin. Elution samples (50 %) were seperated by SDS-PAGE with subsequent commassie staining followed by mass spectrometry analysis.

### Mass spectrometry of BioID samples

For liquid chromatography (LC)–mass spectrometry (MS)/MS analysis, Coomassie-stained lanes of label free BioID samples (BirA*Rab35 vs. Rab35) were cut into each 15 gel pieces per sample and digested in-gel by trypsinization. Peptides were analyzed by a reversed-phase capillary liquid chromatography system (Ultimate 3000 nanoLC system, Thermo Scientific) connected to an Orbitrap Elite mass spectrometer (Thermo Scientific). Identification and quantification of proteins were performed using MaxQuant (version 1.5.2.8) software, searched against the HUMANswiss database (April, 2014) and summarized with the software Scaffold (Proteome Software). The mass tolerance of precursor and sequence ions was set to 10 ppm and 0.35 Da, respectively. Methionine oxidation and the acrylamide modification of cysteine were used as variable modifications. Proteins with at least two detected sequenced peptides (razor unique peptides) with a minimum length of 7 amino acids were counted as biotinylated and specifically bound. False discovery rates were estimated to be <1% based on matches to reversed sequences in the concatenated target-decoy database. Label-free quantification (LFQ)-intensity ratio of BirA*-Rab35- over BirA*-biotinylated proteins was used to rank the detected proteins according to their specific Rab35-association (enrichment). Proteins that associated with BirA*-Rab35 only were considered as “selective” (Supplementary Data 1), proteins with a LFQ-intensity ratio (BirA*-Rab35- over BirA*) >5 as “enriched” (Supplementary Table 1).

### Data and reproducibility

All data are represented as mean ± standard deviation (SD) for *n* = 2 independent experiments or mean ± standard error of the mean (SEM) for *n* > 2 independent experiments or number of animals, and normalized where indicated. GraphPad Prism 5 and ‘R‘ software was used to perform statistical analysis. If not otherwise indicated, normalized data were analyzed using one-sample two-tailed Student’s *t*-tests. For data sets from more than two different genotypes, depicted *p*-values were corrected for multiple testing using Holm’s multiple comparison test^63^. Level of significance are depicted by asterisks in the figures: *p < 0.05, **p < 0.01, ***p < 0.001, ****p < 0.0001. n.s. non-significant. Exact *p*, *t*, and df values are provided in the respective figure legends.

### Reporting summary

Further information on research design is available in the Nature Research Reporting Summary linked to this article.

## Supplementary information

Supplementary Information

Peer Review File

Supplementary Data 1

Supplementary Data 2

Reporting Summary

## Data Availability

The data that support these findings are available from the authors on request. Numerical source data for Figs. 1–10, Supplementary Figs. 1 and 5–8, and uncropped versions of blots and gels for Figs. 1 and 3–9, Supplementary Figs. 1, 4, and 5 are provided in the Source Data File.

## Source data

Source Data
