# Supplementary material for: Rab35-regulated lipid turnover by myotubularins represses mTORC1 activity and controls myelin growth
Source: Nat Commun. 2020 Jun 5;11:2835. doi: 10.1038/s41467-020-16696-6 (PMC7275063; doi:10.1038/s41467-020-16696-6)
Supplement: Supplementary file 1 — Supplementary Information [file 41467_2020_16696_MOESM1_ESM.pdf]

Supplementary File for

Rab35-regulated lipid turnover by myotubularins represses mTORC1 activity and controls myelin growth

Linda Sawade, Federica Grandi, Marianna Mignanelli, Genaro Patiño-López, Kerstin Klinkert, Francina Langa Vives, Roberta Di Guardo, Arnaud Echard, Alessandra Bolino, Volker Haucke

Inventory of supplementary items

Supplementary figures

Supplementary figure 1 | Complex formation of Rab35-GTP with endosomal MTMR lipid phosphatases.

Supplementary figure 2 | Rab35 can recruit MTMR13 to late endosomes/lysosomes.

Supplementary figure 3 | Generation of Schwann cell-specific *Rab35* conditional knockout (cKO^{SC}) mice.

Supplementary figure 4 | mTORC1 hyperactivation upon Rab35, MTMR2, MTMR13 or MTMR5 depletion.

Supplementary figure 5 | Effects of Rab35 loss on the endo-/lysosomal system and on mTORC1 signaling.

Supplementary figure 6 | mTORC1 pathway overactivation in *Rab35* cKO does not alter the onset of myelination.

Supplementary figure 7 | Rescue of mTORC1 hyperactivity induced by loss of Rab35 by pharmacological inhibition of PI 3-phosphate synthesis.

Supplementary figure 8 | Inhibition of mTORC1 activity or PI 3-phosphate synthesis rescues increased myelin protein expression in Schwann cells depleted of Rab35.

Supplementary tables

Supplementary table 1 | Enriched putative Rab35 interactors

Supplementary table 2 | Inhibitors

Supplementary data (available as a separate Excel file)

Supplementary Data 1 | Selective putative Rab35 interactors

Supplementary Data 2 | Plasmids, Primer, Antibodies

Supplementary figures

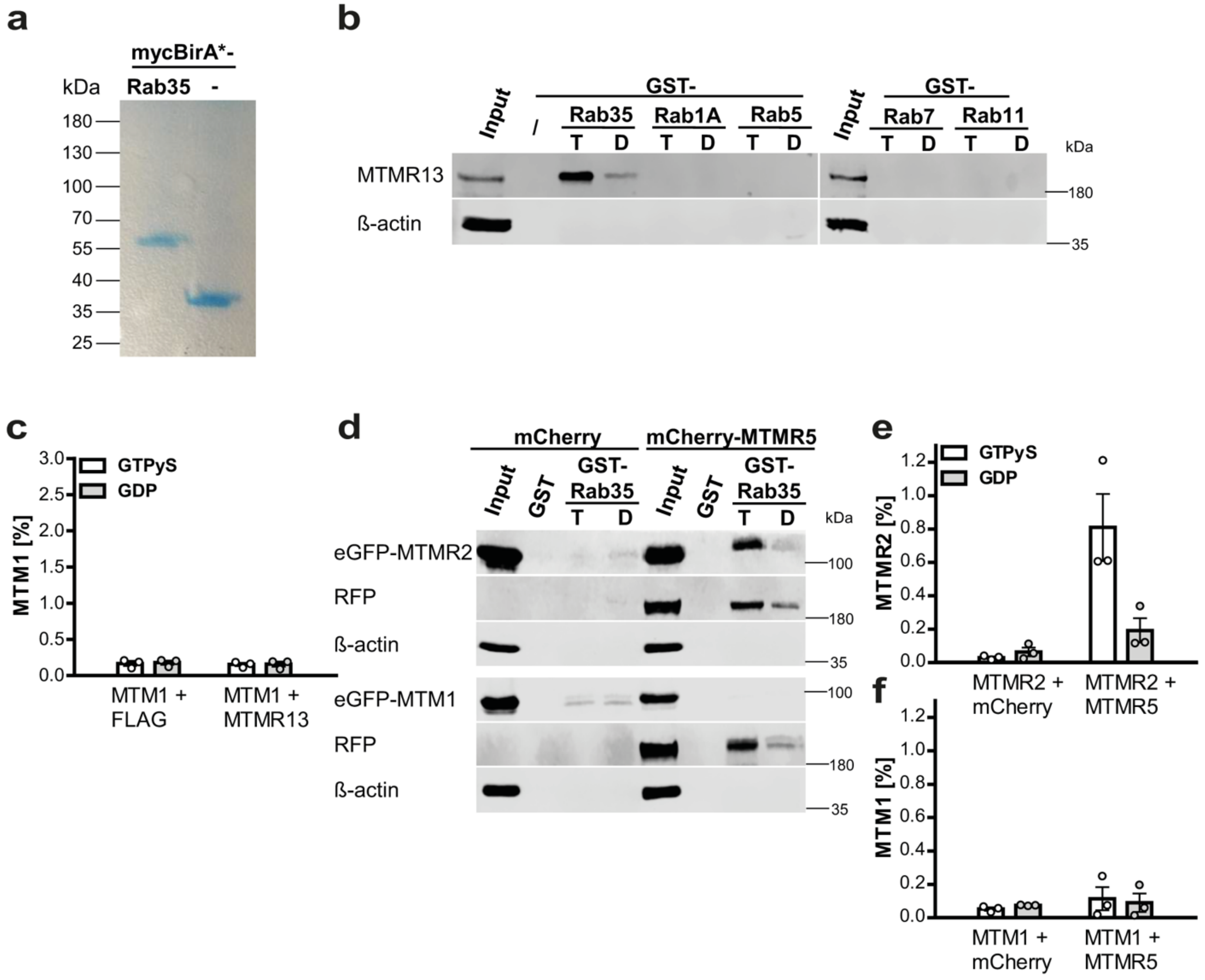

Supplementary Figure 1 | Complex formation of Rab35-GTP with endosomal MTMR lipid phosphatases. (a) related to figure 1a: Representative Coomassie blue stained SDS-PAGE gel of BioID-samples captured by streptavidin from Hek293T cells overexpressing BirA*-Rab35 (left) or BirA* (right) from n = 2 independent experiments. **(b)** MTMR13 specifically associates with Rab35. Affinity chromatography from lysates of Hek293T cells expressing FLAG-MTMR13 cells incubated with nucleotide-loaded GST-Rab35, -Rab1A, Rab5, Rab7 or Rab11. Bound proteins were analyzed by immunoblotting. Representative immunoblot from n = 6 independent experiments is shown. Input: 5 % of total protein added to the assay. T – GTP, D – GDP. **(c)** Active Rab35•GTP does not interact with MTM1 via MTMR13. Quantification of figure 1g: Depicted is the fraction of Rab35-bound MTM1 as % of total input material. Data are from n= 3 independent experiments and represent mean ± SEM. **(d-f)** Active Rab35•GTP interacts with MTMR2 via MTMR5. Lysates from Hek293T cells co-expressing eGFP-MTM1 or -MTMR2 and mCherry-

MTMR5 or mCherry (as a control) were incubated with nucleotide-loaded GST-Rab35. (d) Representative immunoblot for eGFP, RFP, and β-actin as a control. Input: 5 % of total protein added to the assay. (e) Quantification of Rab35-bound MTMR2 as shown in the representative immunoblot in d. The total fraction of bound protein relative to the input is shown. Data represent mean ± SEM from n=3 independent experiments. (f) Quantification of Rab35-bound MTM1. Data represent mean ± SEM from n=3 independent experiments.

Numerical source data and unprocessed blots are reported in the Source Data file.

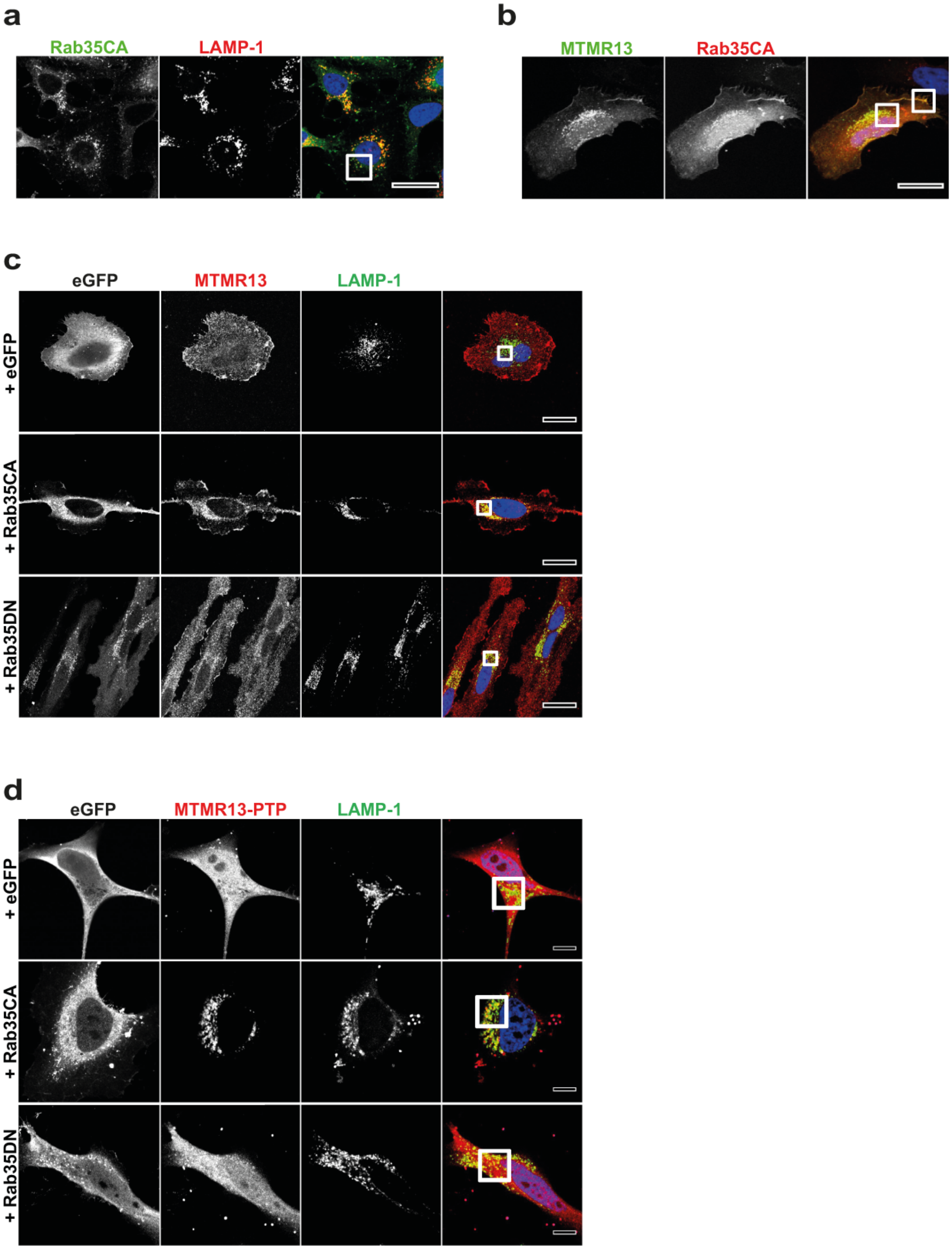

Supplementary Figure 2 | Rab35 can recruit MTMR13 to late endosomes/ lysosomes. (a, b) related to figure 2b, c: Scale bars, 30 µm (a) Lower magnification of figure 2b (white square). n =

2 independent experiments. (b) Lower magnification of figure 2c (white squares). n = 5 independent experiments. **(c, d)** related to figure 2d-g: eGFP is shown in grey only. (c) Lower magnifications of figure 2d (white squares). Scale bars, 30 µm. n = 24 (Rab35CA) and 29 (Rab35DN) cells in 4 independent experiments. (d) Lower magnifications of figure 2f (white squares). Scale bars, 10 µm. n = 32 (Rab35CA) and 31 (Rab35DN) cells in 4 independent experiments.

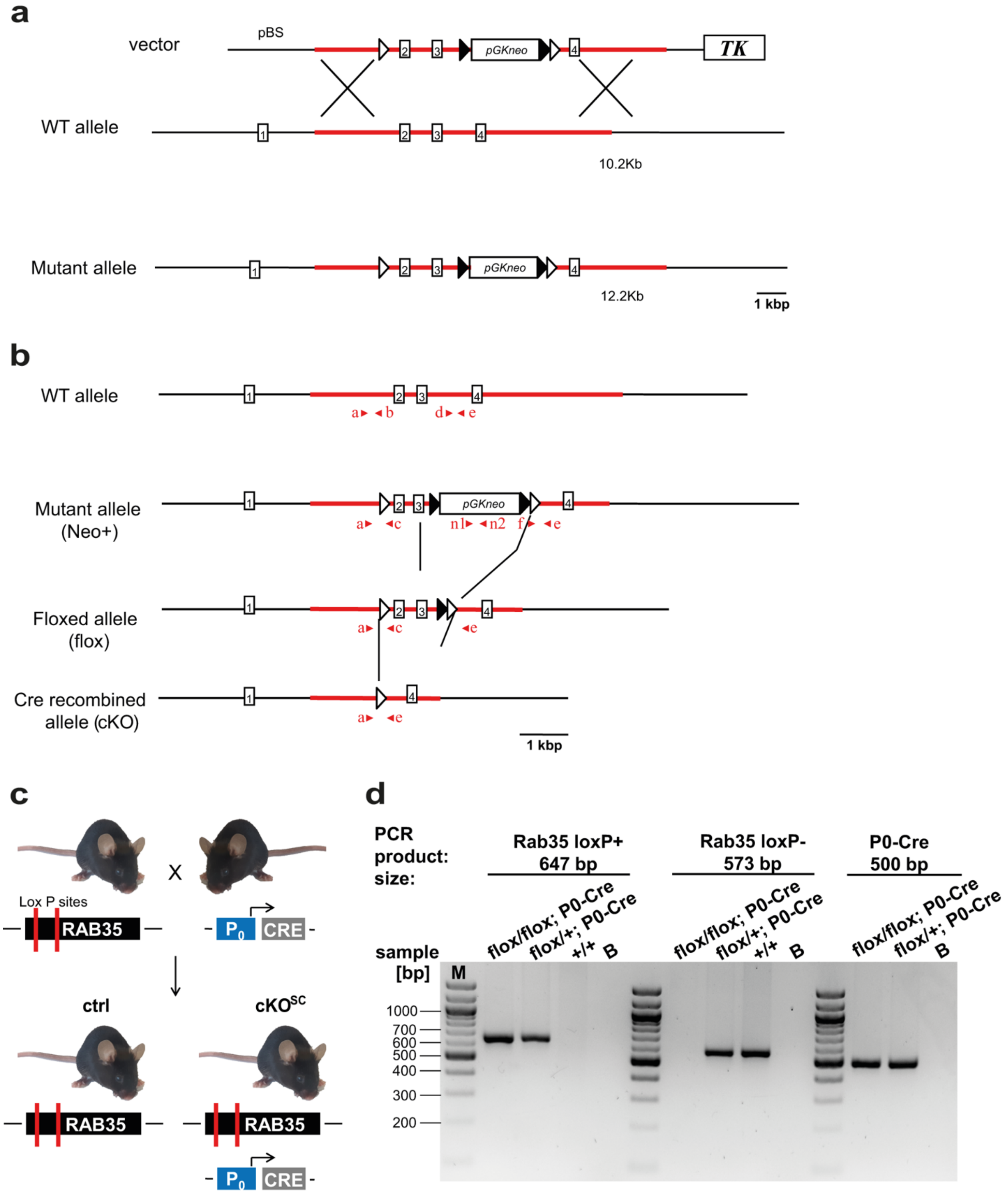

Supplementary Figure 3 | Generation of Schwann cell-specific *Rab35* conditional knockout (cKO^{SC}) mice. (a, b) Generation of floxed *Rab35* (*Rab35$^{flox/flox}$*) mice for conditional gene knockout. (a) Schematic diagram of the targeting vector (top) containing the manipulated mouse

genomic *Rab35* locus region used for incorporation into ES cells. Homologous recombination with the *Rab35* wild type (WT) (below) results in the production of a mutant allele (bottom), in which the second and third coding exons are flanked by loxP recombination sites (white triangles) and a neomycin selection cassette (pGKneo). The Neomycin cassette is flanked by flippase (flp) recognition sites (FRT; black triangles). TK - thymidine kinase (b) From the top: Schematic diagram of the wild-type allele (WT) containing exon 2, 3 and 4 in the designated manipulated region. Below the mutant allele containing the Neomycin cassette is depicted. Flippase-FRT-mediated recombination removes the Neomycin cassette and results in a floxed (flox) allele. Cre-mediated recombination removes exon 2 and exon 3 to generate a knockout allele (cKO). PCR-Primers used to identify the different alleles are indicated in red: 'a' - Lox5F, 'b' - Lox5R, 'c' - PL452-LoxP-Sc1R, 'd' - Frt 3F, 'e' - Frt 3R, 'f' - PL452_3'-Sc1F, 'n1' - NeoF, 'n2' - NeoR targeting the Neomycin cassette to identify the mutant allele (Neo^{+}). **(c)** Schwann cell specific *Rab35* knockout (cKO^{SC}) mice were generated by crossing *Rab35*$^{flox/flox}$mice with transgenic mice that encode a Cre recombinase under control of the Schwann cell specific *P0-Cre* promoter (*P0-Cre*). Breeding of heterozygous *Rab35*$^{flox/-}$ x P0-Cre mice yielded homozygous *Rab35*$^{flox/flox}$x *P0-Cre* mice. The latter were bred with *Rab35*$^{flox/flox}$mice. *Rab35* WT – *Rab35*$^{flox/flox}$; *Rab35* KO^{SC} (*Rab35*$^{flox/flox}$x *P0-Cre*). **(d)** PCR-amplification and products used to identify the genotypes for breeding and preparation and verified for all animals used in this study, with DNA obtained from mice biopsies. Amplified DNA-fragment from the *Rab35* gene without (left; l*ox*P+) or with (middle; *lox*P-) *lox*P-sites. Right: Amplified DNA-fragment from P0-Cre locus in Cre-positive animals. M – molecular weight marker lane.

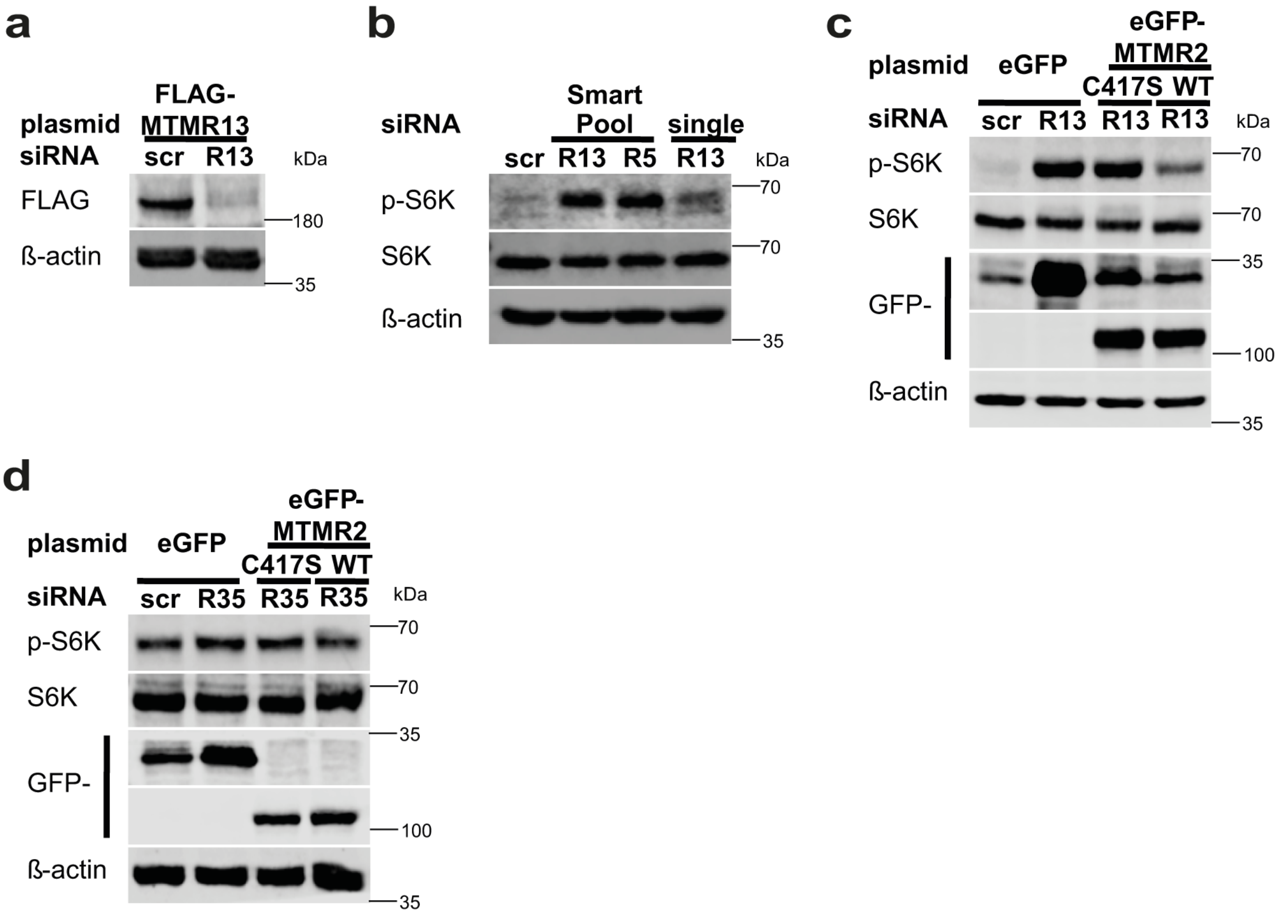

Supplementary Figure 4 | mTORC1 hyperactivation upon Rab35, MTMR2, MTMR13 or MTMR5 depletion. **(a)** SiRNA-mediated depletion of MTMR13. HEK293T cells were cotransfected with siRNA targeting MTMR13 and a plasmid encoding FLAG-MTRM13. Lysates were analyzed by immunoblotting for FLAG and ß-actin as loading control. Scrambled siRNA (scr) transfected cells were used as a control. n = 2 independent experiments. **(b)** Depletion of MTMR13 or MTM5 by siRNA SmartPools or a single siRNA causes mTORC1 hyperactivation. HEK293T cells were treated with Smart Pool siRNAs targeting MTMR13 or MTMR5, a specific siRNA targeting MTMR13, or scrambled siRNA (scr) as a control. p-S6K and total S6K protein level were analyzed by immunoblotting as a readout for mTORC1 activation. mTORC1 hyperactivation was more pronounced in SmartPool siRNA-treated cells. n = 2 independent experiments. **(c, d)** Partial rescue of mTORC1 hyperactivation in cells depleted of Rab35 (c) or MTMR13 by overexpression of active wild-type MTMR2. HEK293T cells treated with scrambled siRNA (scr), smartPool siRNA targeting MTMR13 (reproduced in n = 2 independent experiments for active MTMR2 and single-targeting siRNA against MTMR5 or MTMR13) (c) or with specific siRNA targeting Rab35 (reproduced in n = 4 independent experiments) (d) were transfected with

plasmids encoding eGFP, siRNA-resistant mouse eGFP-MTMR2 (WT) or phosphatase-inactive mouse MTMR2 (C417S) and analyzed by immunoblotting for the indicated proteins. Overexpressed inactive mutant MTMR2 was less effective at rescuing mTORC1 hyperactivity upon depletion of Rab35 or MTMR13.

Unprocessed blots are reported in the Source Data file.

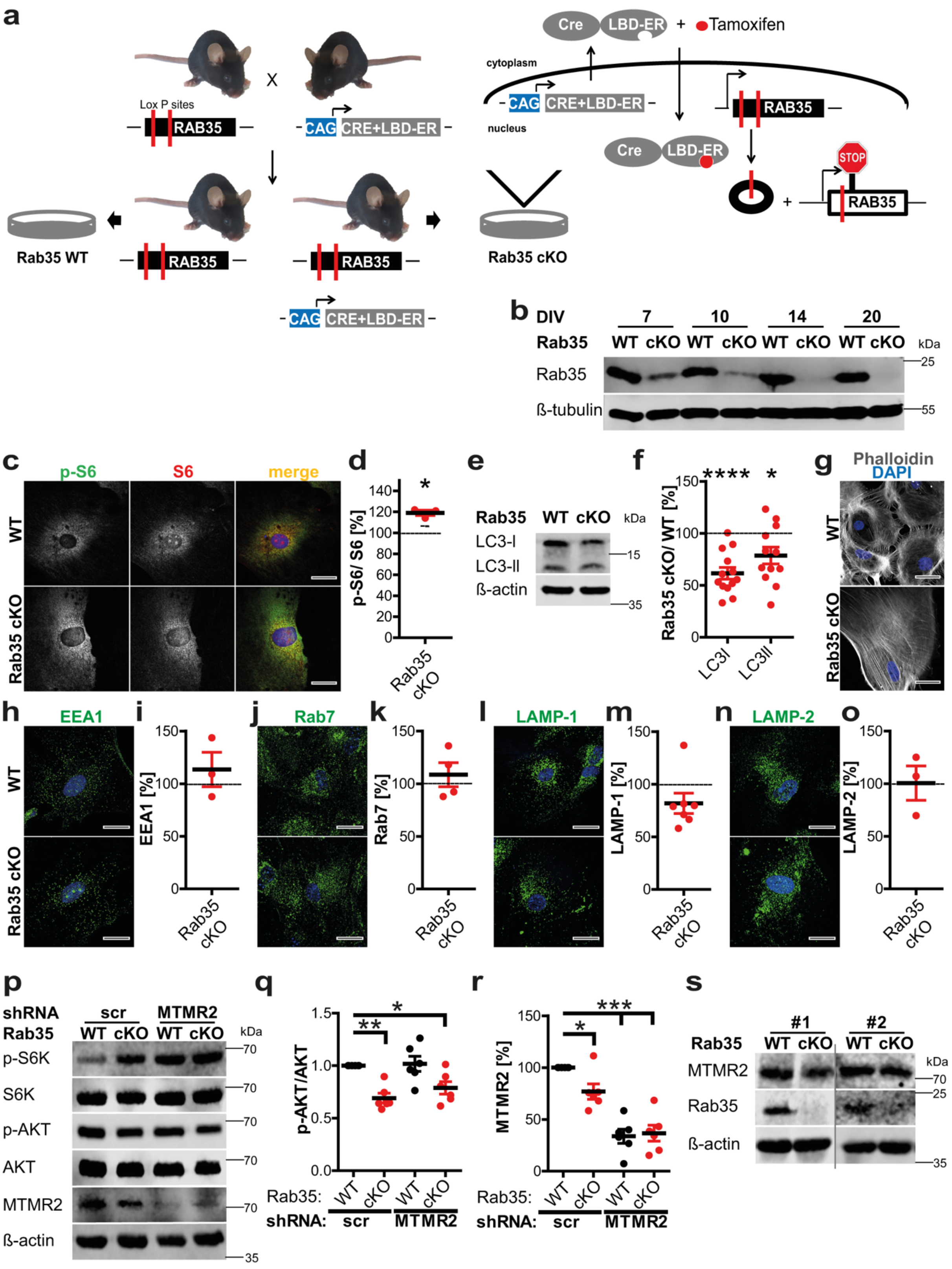

Supplementary Figure 5 | Effects of Rab35 loss on the endo-/lysosomal system and on mTORC1 signaling. (a, b) Tamoxifen-induced conditional KO of Rab35. (a) Scheme for the

generation of tamoxifen inducible Rab35 knockout mice or cells. *Rab35$^{flox/flox}$* mice were crossed with *CAG-Cre*^{ER} mice, which express Cre^{ER} recombinase under *CAG* promoter. Cre^{ER} is ubiquitously expressed and recombination can be induced by tamoxifen application. Breeding of heterozygous *Rab35$^{flox/-}$* x *CAG-Cre*^{ER} mice yielded homozygous *Rab35$^{flox/flox}$* x *CAG-Cre*^{ER} mice. The latter were bred with *Rab35$^{flox/flox}$* mice. (b) Astrocyte cultures were prepared from littermate *Rab35* cKO (*Rab35$^{flox/flox}$* x *CAG-Cre*^{ER}) and *Rab35* WT (*Rab35$^{flox/flox}$*) animals and supplemented with tamoxifen to yield *Rab35* WT (*Rab35$^{flox/flox}$*) and cKO (*Rab35$^{flox/flox}$* x *CAG-Cre*^{ER}) cultures from day *in vitro* (DIV) 0 on. Rab35 loss after different days in culture was confirmed by immunoblotting of lysates. ß-tubulin was analyzed as a loading control. n = 1 independent experiment for DIV7, 14, 10; DIV20: lysates for all quantifications were prepared from DIV20-22 cultures and verified for Rab35 depletion. **(c, d)** mTORC1 is hyperactive in *Rab35* cKO astrocytes. (c) Confocal images of *Rab35* WT (*Rab35$^{flox/flox}$*) and cKO (*Rab35$^{flox/flox}$* x *CAG-Cre*^{ER}) astrocytes in culture (DIV20) immunostained for phosphorylated ribosomal protein S6 (pS6) and total S6. DAPI (blue) was used to visualize cell nuclei. Scale bar, 30 µm (d) Quantification of representative data shown in c. Mean intensities of p-S6 and S6 signals were measured in single cells masked by fluorophore-conjugated Phalloidin labelling. p-S6/ S6 ratio in WT was set to 1. Data are from n = 3 independent experiments. One sample two-tailed student's t-test with a theoretical mean of 100; $^{*}p = 0.0171$, $t = 7.55$, $df = 2$. **(e, f)** Decreased steady-state levels of LC3 in *Rab35* cKO astrocytes. (e) Immunoblot analysis of lysates from *Rab35* WT (*Rab35$^{flox/flox}$*) and cKO (*Rab35$^{flox/flox}$* x *CAG-Cre*^{ER}) astrocytes using antibodies against LC3I/II. ß-actin was used as a loading control. (f) Quantification of representative data shown in e. LC3 signals were normalized to the loading control and plotted as a fraction of *Rab35* WT LC3. One sample two-tailed student's t-test with a theoretical mean of 100. LC3-I: n = 13 independent experiments, $p < 0.0001$, $t = 6.81$, $df = 12$; LC3-II: n = 12 independent experiments, $^{*}p = 0.0221$, $t = 2.661$, $df = 11$. **(g)** related to figure 5e: Increased size of Rab35 cKO astrocytes. WT and cKO astrocytes fixed at DIV20 were labelled by Phalloidin-AlexaFluor568 (grey) and analyzed by confocal imaging to quantitatively determine the cell area using Phalloidin as a mask. DAPI (blue) was used to visualize cell nuclei. **(h-o)** Rab35 loss does not cause major alterations in the endo-/lysosomal system. Representative confocal images and quantitative analyses of the steady-state levels of (h, i) early endosomal antigen 1 (EEA1), (j, k) late endosomal Rab7, (l, m) late endosomal/ lysosomal LAMP-1, and (n, o) the lysosomal marker LAMP-2 (green). Cell nuclei were visualized by DAPI (blue). Sum intensities were normalized to cell area marked by fluorophore-conjugated Phalloidin labelling, and depicted

as the fraction of *Rab35* cKO (*Rab35*$^{flox/flox}$ x *CAG-Cre*^{ER}) to WT (*Rab35*$^{flox/flox}$) signals. Scale bars, 30 µm. One sample two-tailed student's t-test with a theoretical mean of 100; EEA1: n = 3 independent experiments, p = 0.4956, t = 0.8261, df = 2; Rab7: n = 4 independent experiments, p = 0.5061, t = 0.7532, df = 3; LAMP-1: n = 7 independent experiments, p = 0.1104, t = 1.872, df = 6; LAMP-2: n = 3 independent experiments, p = 0.9769, t = 0.03269, df = 2. **(p-r)** Related to figure 5f: Depletion of MTMR2 in astrocytes causes mTORC1 hyperactivity. (p) Lysates from lentivirally transduced Rab35 WT (*Rab35*$^{flox/flox}$) or cKO (*Rab35*$^{flox/flox}$ x *CAG-Cre*^{ER}) astrocytes expressing shRNA-encoding lentiviruses targeting MTMR2 or non-targeting scrambled (scr) control shRNA were analyzed by immunoblotting for p-S6K1, total S6K1, p-AKT, total AKT, MTMR2 and ß-actin (as a loading control). (q) Quantification of representative data shown in p. Depicted is the p-AKT/ AKT ratio normalized to WT/ scrambled shRNA (scr) control. Data are from n = 6 independent experiments. One sample two-tailed student's t-test with a theoretical mean of 1, followed by Holm's Multiple Comparison Test to correct for multiple testing; cKO + scr: **p = 0.0033, t = 6.72285, df = 5; WT + shMTMR2: p = 0.8170, t = 0.24388, df = 5; cKO + shMTMR2: *p = 0.033, t = 3.54479, df = 5. (r) Efficacy of MTMR2 knockdown. Depicted are MTMR2 levels normalized to ß-actin as a loading control. Values for WT/ scrambled shRNA controls were set to 100. Data are from n = 6 independent experiments. One sample two-tailed student's t-test with a theoretical mean of 100 followed by Holm's Multiple Comparison Test to correct for multiple testing: cKO + scr: *p = 0.0261, t = 3.12641, df = 5; WT + shMTMR2: ***p = 0.0006, t = 9.68319, df = 5; cKO + shMTMR2: ***p = 0.0009, t = 8.21648, df = 5. **(s)** Related to figure 5g: Reduced MTMR2 protein levels in *Rab35* cKO (*Rab35*$^{flox/flox}$ x *CAG-Cre*^{ER}) astrocytes in culture. Representative immunoblots for MTMR2 and Rab35 from two independent experiments. ß-actin was used as a loading control.

All data represent mean ± SEM. Numerical source data and unprocessed blots are reported in the Source Data file.

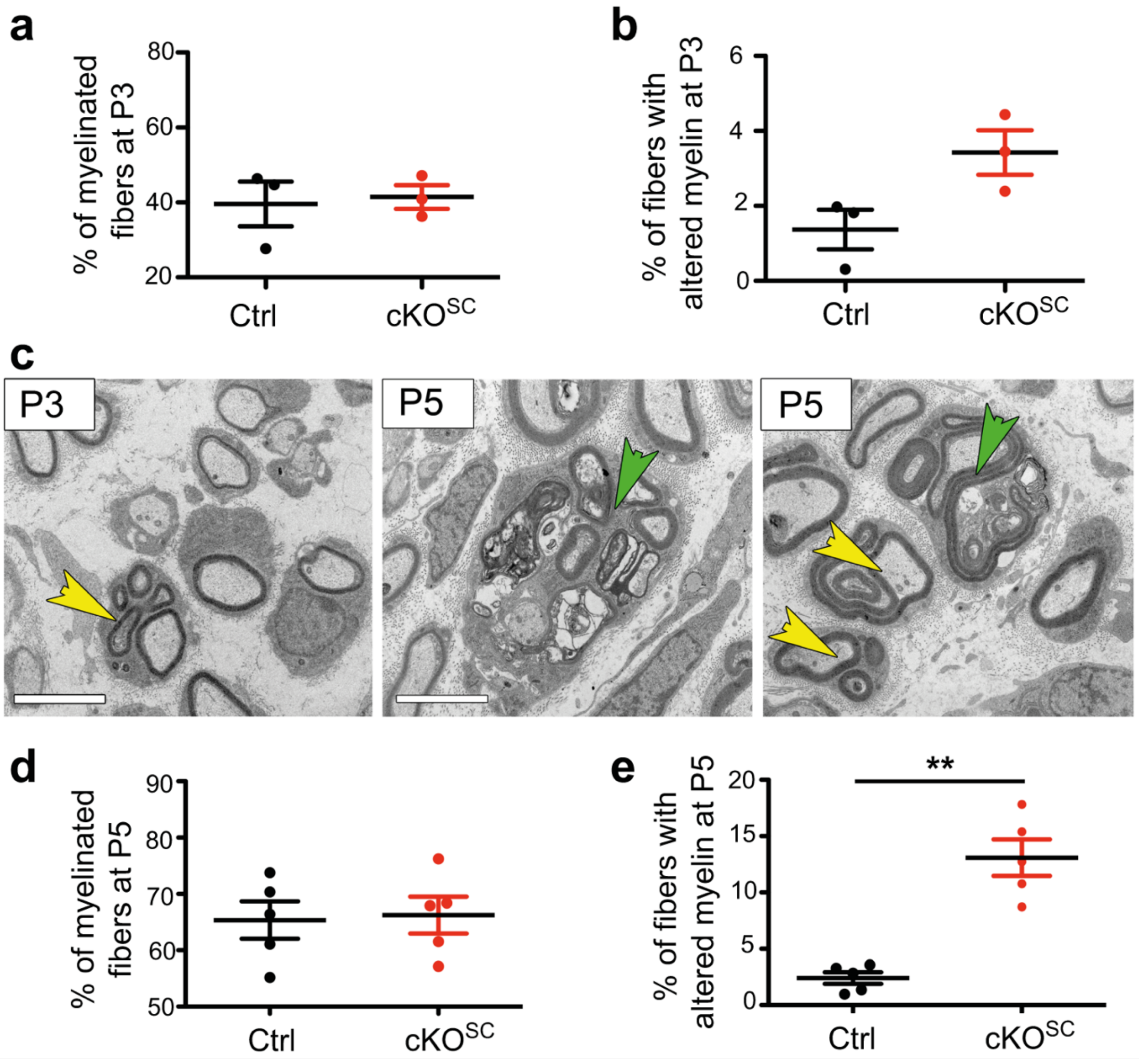

Supplementary Figure 6 | mTORC1 pathway overactivation in *Rab35* cKO does not alter the onset of myelination. (a) Ultrastructural analysis of sciatic nerve fibers at P3 and quantification of the fraction of myelinated fibers relative to the total number of fibers: control (*Rab35* $^{flox/flox}$) 39.6% ± 5.97 and *Rab35* cKO 41.46% ± 3.16, n = 849 and n = 731 fibers, respectively, n = 3 animals per genotype, p >0.9999, two-tailed Mann-Whitney t-test. **(b)** Ultrastructural analysis of sciatic nerve fibers at P3 and quantification of the percentage of altered fibers carrying myelin degeneration or comma shape/recurrent loops on the total number of fibers: control (*Rab35* $^{flox/flox}$) 1.37% ± 0.53 and *Rab35* cKO 3.43% ± 0.59, n = 849 and n =731 fibers, respectively, n = 3 animals per genotype; p = 0.1000, two-tailed Mann-Whitney t-test. Note that altered fibers in control correspond to comma shaped structures that are typically observed at early stages of development in the mouse PNS. **(c)** Representative images of ultrastructural analysis of control and *Rab35*

cKO^{SC} sciatic nerves at P3 and P5, quantified in a,b and d,e. Yellow arrowheads mark recurrent loops and comma shape, green arrowheads mark myelin degeneration. Scale bar, 3.4 µm. **(d)** Ultrastructural analysis of sciatic nerve fibers at P5 and quantification of the fraction of myelinated fibers relative to the total number of fibers: control (*Rab35$^{flox/flox}$*) 65.36% ± 3.31 and *Rab35* cKO 66.24% ± 3.26, n = 1617 and n = 1663 fibers, respectively, n = 5 animals per genotype, p = 0.84, two-tailed Mann-Whitney t-test. **(e)** Ultrastructural analysis of sciatic nerve fibers at P5 and quantification of the percentage of altered fibers carrying myelin degeneration or comma shape/recurrent loops on the total number of fibers: control (*Rab35$^{flox/flox}$*) 2.4% ± 0.52 and *Rab35* cKO 13.1% ± 1.62, n = 1617 and n =1663 fibers, respectively, n = 5 animals per genotype; **p = 0.0079, two-tailed Mann-Whitney t-test.

All data represent mean ± SEM. Numerical source data are reported in the Source Data file.

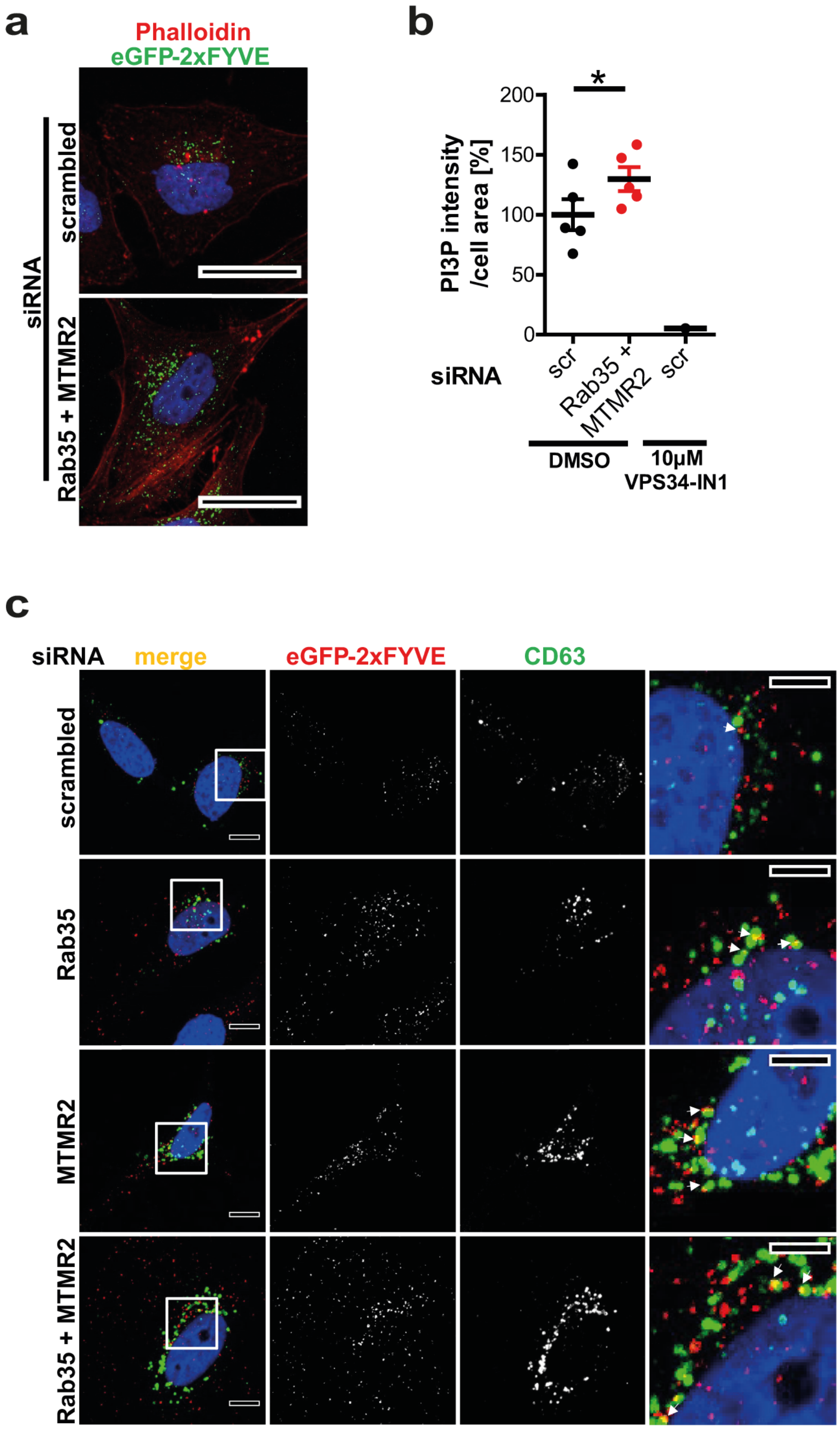

Supplementary Figure 7 | Rescue of mTORC1 hyperactivity induced by loss of Rab35 by pharmacological inhibition of PI 3-phosphate synthesis. (a, b) Co-depletion of Rab35 and

MTMR2 in HeLa cells results in increased levels of PI(3)P. HeLa cells were treated with non-targeting scrambled (scr) control siRNA or siRNAs specific for Rab35 and MTMR2. (a) Representative confocal images of siRNA transfected HeLa cells stained for PI(3)P using recombinant eGFP-2xFYVE as a probe (green) and for F-actin by fluorescently-labelled phalloidin (red). Blue, DAPI-stained nuclei. Scale bars, 30 µm. (b) Quantification of representative data shown in a. The sum intensity of eGFP-positive puncta normalized to the cell area and control conditions (scr +DMSO). Two-tailed paired student's t-test. Data are from n = 5 independent experiments and represent mean ± SEM. *p = 0.0366, t = 2.414, df = 4. PI(3)P levels in HeLa cells treated for 1 h with 10 µM the class III PI 3-kinase inhibitor VPS34-IN1 were quantified as a control for specificity. **(c)** Representative confocal images from n = 5 independent experiments pertaining to figure 9c: HeLa cells, treated with scrambled (scr), Rab35, MTMR2, or Rab35 and MTMR2 targeting siRNA, were immunostained for PI(3)P (red) and CD63 (green). Cell nuclei were visualized with DAPI (blue). Arrows indicate colocalization. Scale bars, 10 µm (left), 5 µm (right).

Numerical source data are reported in the Source Data file.

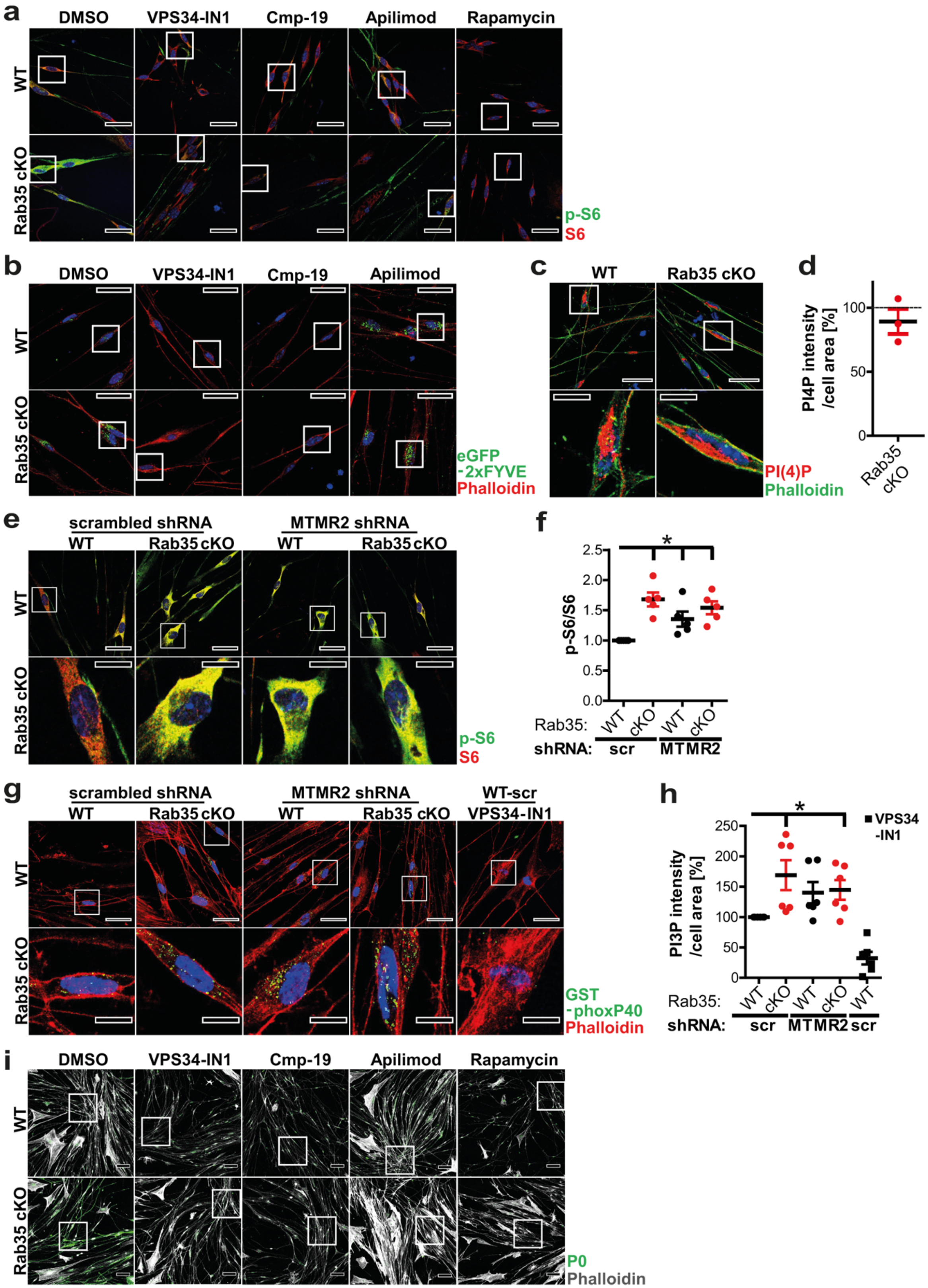

Supplementary Figure 8 | Repression of mTORC1 activity or PI 3-phosphate synthesis rescues increased myelin protein expression in Schwann cells depleted of Rab35. (a) mTORC1

hyperactivity in differentiated *Rab35* cKO Schwann cells is rescued by inhibition of VPS34 or the PI(3)P 5-kinase PIKFYVE. Low magnification views of representative confocal images shown in figure 10a (depicted as white squares) of *Rab35* WT and cKO Schwann cells treated with DMSO or the indicated inhibitors immunostained for p-S6 (green) and total S6 (red). Cell nuclei were visualized with DAPI (blue). Scale bars, 30 µm. n = 6 (DMSO), 4 (Apilimod, Cmp-19) and 3 (VPS34-IN1, Rapamycin) independent experiments. **(b)** Depletion of Rab35 or inhibition of VPS34 or PIKFYVE results in increased PI(3)P levels in Schwann cells in culture. Low magnification views of representative confocal images shown in figure 10c (depicted as white squares) of *Rab35* WT and cKO Schwann cells treated with DMSO or the indicated inhibitors and immunostained for PI(3)P (green) and Phalloidin (red) . Cell nuclei were visualized with DAPI (blue). Scale bars, 30 µm. n = 4 (DMSO) and 3 (VPS34-IN1, Cmp-19, Apilimod) independent experiments. **(c, d)** Rab35 cKO does not alter the levels of PI(4)P in Schwann cells. (c) Representative confocal images of *Rab35* WT (*Rab35$^{flox/flox}$*) or cKO Schwann cells in culture immunostained with antibodies Against PI(4)P (red) and for F-actin by fluorescently-labelled phalloidin (green). Cell nuclei were visualized with DAPI (blue). Lower images are high magnifications of the image areas depicted as white squares above. Scale bars, upper: 30 µm, lower: 10 µm. (d) Quantification of representative data shown in c. The sum intensity of PI(4)P-positive puncta in Schwann cell somata normalized to the somata area determined by phalloidin staining is depicted. Values from *Rab35* WT Schwann cells were set to 100%. Data are from n=3 independent experiments. One sample two-tailed student's t-test with a theoretical mean of 100 was used. p = 0.3854, t = 1.102, df = 2. **(e-h)** Loss of MTMR2 exacerbates mTORC1 hyperactivity and PI(3)P levels in *Rab35* cKO Schwann cells. Primary Schwann cells were transduced with lentiviruses encoding scrambled (scr) or anti-MTMR2 shRNA. Fixed cells were immunostained and analyzed by confocal imaging. Cell nuclei were visualized with DAPI (blue). Lower images are high magnifications of the image areas depicted as white squares above. Scale bars, upper: 30 µm, lower: 10 µm. (e) Representative confocal images of primary Schwann cells immunostained for p-S6 (green) and total S6 (red) as a readout for mTORC1 activity. (f) Quantification of representative data shown in e. The normalized p-S6 to S6 mean intensity ratio in cell somata is plotted. Values for WT Schwann cells treated with scrambled shRNA were set to 1. Data are from n = 5 independent experiments, and were analyzed by one sample two-tailed student's t-test with theoretical means of 1, for all conditions normalized to control (WT + scr), followed by Holm's Multiple Comparison Test to correct for multiple testing; cKO + scr: *p = 0.0122, t = 5.9295, df =

4; WT + shMTMR2: *p = 0.0473, t = 2.8317, df = 4; cKO + shMTMR2: *p = 0.0149, t = 5.0121, df = 4. (g) Representative confocal images of transduced *Rab35* WT or cKO Schwann cells in culture immunostained for PI(3)P using GST-phoxP40 (green) as a probe and for F-actin by fluorescently-labelled phalloidin (red). (h) Quantification of representative data shown in g. The sum intensity of GST-positive puncta normalized to the cell area and *Rab35* WT Schwann cells transduced with scrambled shRNA were set to 100 %. Acute application of 10µM VPS34IN1 for 1h prior to fixation reduced GST-positive puncta (used as a control). Data are from n = 6 independent experiments, and were analyzed by one sample two-tailed student's t-test with theoretical means of 100, for all conditions normalized to control (WT + scr); cKO + scr: *p = 0.0379, t = 2.801, df = 5; WT + shMTMR2: p = 0.0694, t = 2.304, df = 5; cKO + shMTMR2: *p = 0.0408, t = 2.740, df = 5; WT + scr + VPS34-IN1: **p = 0.0012, t = 6.546, df = 5. **(i)** Increased myelin protein expression in *Rab35* cKO Schwann cell cultures is rescued by inhibition of mTORC1, VPS34 or PIKFYVE. Low magnification views of representative confocal images shown in figure 10e (depicted as white squares) of *Rab35* WT (*Rab35$^{flox/flox}$*) or cKO Schwann cells treated with DMSO or the indicated inhibitors and immunostained for P0 (green) and F-actin (grey). Scale bars, 100 µm. n = 8 (DMSO), 6 (Apilimod, Cmp-19), 5 (VPS34-IN1) and 4 (Rapamycin) independent experiments. All data represent mean ± SEM. Numerical source data are reported in the Source Data file.

Supplementary table 1 | Rab35-associated proteins enriched more than fivefold (LFQ-intensity BirA*Rab35/BirA*) in BioID

n = 2 independent experiments. Enrichment: mean ± SD; bold — linked to Rab35 by literature

Rank	Gene	Protein	Fold enrichment
1	RAB35	Ras-related protein Rab-35	745.72 ± 347.31
**2**	**TFRC**	**Transferrin receptor protein 1**	**45.86 ± 48.09**
3	SLC3A2	4F2 cell-surface antigen heavy chain	44.72 ± 35.72
4	LSR	Lipolysis-stimulated lipoprotein receptor	36.04 ± 11.34
5	SLC12A2	Solute carrier family 12 member 2	36.00 ± 5.00
6	STX12	Syntaxin-12	35.95 ± 38.94
7	CACHD1	VWFA and cache domain-containing protein 1	34.44 ± 39.70
**8**	**SBF1**	**Myotubularin-related protein 5**	**28.92 ± 1.45**
**9**	**CHML**	**Rab proteins geranylgeranyltransferase component A 2**	**23.35 ± 16.53**
10	SLC1A5	Neutral amino acid transporter B(0)	22.19 ± 5.44
11	CNNM3	Metal transporter CNNM3	21.10 ± 18.18
12	ATP2B1	Plasma membrane calcium-transporting ATPase 1	13.35 ± 4.94
13	UACA	Uveal autoantigen with coiled-coil domains and ankyrin repeats	10.90 ± 6.91
14	IGF2R	Cation-independent mannose-6-phosphate receptor	8.17 ± 1.84
15	SLC4A7	Sodium bicarbonate cotransporter 3	7.01 ± 1.09
16	MARCKS	Myristoylated alanine-rich C-kinase substrate	6.65 ± 0.02
17	VAMP2;VAMP3	Vesicle-associated membrane protein 2	6.53 ± 2.11

Supplementary table 2 | Concentration of stock solutions after dissolving in DMSO and sources of used inhibitors.

**Inhibitor**	**Target protein**	**Stock solution**	**Source**
Apilimod	PIKFyve	25 mM	BioVision, B1129
Compound-19	VPS34	10 mM	Selleckchem, S8456
Rapamycin	mTORC1-component FKBP12	2 mM	Santa Cruz, sc3504
SAR405	VPS34	10 mM	kind gift from Sprint Bioscience, Huddinge, Sweden
VPS34-IN1	VPS34	10 mM	provided by Dr. James Hastie (MRC PPU Reagents, Dundee)
