# Supplementary material for: Rab35-regulated lipid turnover by myotubularins represses mTORC1 activity and controls myelin growth
Source: Nat Commun. 2020 Jun 5;11:2835. doi: 10.1038/s41467-020-16696-6 (PMC7275063; doi:10.1038/s41467-020-16696-6)
Supplement: Supplementary file 2 — Peer Review File [file 41467_2020_16696_MOESM2_ESM.docx]

Peer Review Information

**Journal:** Nature Communications

**Manuscript title:** Rab35-regulated lipid turnover by myotubularins represses mTORC1 activity and controls myelin growth

**Corresponding author name(s):** Dr Volker Haucke

**Editorial notes:**

**Transferred manuscripts**

This manuscript has been previously reviewed at another journal that is not operating a transparent peer review scheme. This document only contains reviewer comments and rebuttal letters for versions considered at *Nature Communications*.

**Transferred manuscripts (no peer review at *Nature Communications*)**

This manuscript has been previously reviewed at another journal that is not operating a transparent peer review scheme. The manuscript was considered suitable for publication without further review at *Nature Communications*.

**Redactions – transferred manuscripts (mention of previous referee reports from elsewhere)**

This manuscript has been previously reviewed at another journal that is not operating a transparent peer review scheme. This document only contains reviewer comments and rebuttal letters for versions considered at *Nature Communications*. Mentions of prior referee reports have been redacted.

**Redactions – transferred manuscripts (mention of the other journal)**

This manuscript has been previously reviewed at another journal that is not operating a transparent peer review scheme. This document only contains reviewer comments and rebuttal letters for versions considered at *Nature Communications*. Mentions of the other journal have been redacted.

**Redactions – unpublished data**

Parts of this Peer Review File have been redacted as indicated to maintain the confidentiality of unpublished data.

**Redactions – confidential patient information**

Parts of this Peer Review File have been redacted as indicated to maintain patient confidentiality.

**Redactions – published data**

Parts of this Peer Review File have been redacted as indicated to remove third-party material where no permission to publish could be obtained.

**Amendments – published data**

[Figure X/The figure on page X] in this Peer Review File has been amended to remove third-party material where no permission to publish could be obtained.

**Published data (credit line for image)**

[Figure X/The figure on page X] in this Peer Review File is reproduced with permission from [Copyright Holder].

**Redactions – reviewer opt-out**

Parts of this Peer Review File have been redacted as indicated as we could not obtain permission to publish the reports of reviewer ###.

**Reviewer comments in marked-up manuscript**

In their review of the [first/second/third/...] version of this manuscript, reviewer ### added their comments to the manuscript file. These comments, excluding minor textual revisions, have been copied into this Peer Review File.

**Reviewer comments & decisions:**

**Reviewer comments, first version:**

Reviewers' comments:

Reviewer #1 (Remarks to the Author):

In this paper Rab35 was identified as regulator of myelin growth in the peripheral nervous system by its association with the PI3-phosphatases MTMR13 and MTMR2. The authors how that the regulation occurs by mTOR signaling, thereby linking Rab35 to the known function of MTMR13 and MTMR2 to the AKT-mTORC2 signaling pathway. To arrive at this conclusion the authors use a proteome approach to identify proteins associated with Rab35-GTP followed by biochemical and cell biological assays to validate the effector molecules. In addition, a series of in vivo experiments were conducted to show the function of Rab35 deletion in myelin growth in the peripheral nervous system. The main advance of this paper is to provide a novel function of Rab35 in the nervous system. The observations are intriguing and for most parts the results are convincingly shown. However, there are a number of experiments required to fully complete the study:

1. The paper jumps back and forth from Schwann cells to astrocytes. Since the in vivo function of Rab35 is shown for Schwann cells, the key experiments need to be conducted in these cells and the astrocyte data could be moved to the supplement to avoid redundancy.
2. The peripheral nervous system myelin phenotype needs to be clarified. Figure 2 shows normal g-ratios and overgrowth profiles. However, the co-culture data in Figure 4 suggest hypomyelination. The authors should extend their study to analyze whether hypomyelination is also seen in vivo in mice. Early time points should be included to determine whether the onset is normal.
3. The impact of the paper would increase if the authors could study the physiological role of Rab35 during myelin development in peripheral nervous system. It is for example conceivable that Rab35 activity is important to control the termination of myelin formation. Is the activity of Rab35 altered when myelin growth comes to an end?

Specific comments:
Figure 1: Panel d and e-g should be quantified.
Figure 4: Control groups are missing and need to be provided. In e and g the WT (NT) and WT (rapamycin) are not shown.
Figure 5: the images corresponding to c should be shown.
Figure 6: the images shown in e are of low quality. It is not possible to see any signal.
Supp Fig3: the images corresponding to g should be shown as an overview image
Supp FigS4: the rescue experiments using rapamycin are critical, but only shown in Supp figure, possible because the rescue was only partially successful. The authors should shift these experiments into the main figure and display them as shown in Figure 2e.
Legends: the figure legends are too long and difficult to read. The authors should improve this to make the paper easier to read.


Reviewer #2 (Remarks to the Author):

The authors here explore novel roles for Rab35 GTPase to regulate and connect together several previously known mediators of peripheral axonal myelination and health — specific MTMR phosphatases and mTORC1 kinase activity. Using BioID in HEK293T cells (and as previously reported for conserved proteins in a proteomic study by Gillingham et al), the authors identified Rab35 interactions with inactive phosphatases, MTMR13 and MTMR5. These interactions were defined by preferred binding to GTP-bound Rab35 and validated in HeLa cells. Conditional knockouts of Rab35 in mice revealed a phenocopy to MTMR13 and MTMR2 mutations previously shown with defective Schwann cells in models of CMT4B neuropathy disease. The authors explore and demonstrate a link between Rab35 and MTMR2 functions needed for downregulation of mTORC1 activity in cell cultures and in vivo, bridging previous individual findings on myelination roles for MTMR2 and mTORC1. Epistasis results support the pathway model by showing rescue of Rab35 and MTMR2 mutant mTORC1 activity with either wild type MTMR2 overexpression or inhibition of mTORC1 activity. Given the endosomal/lysosomal membrane and signaling roles already known for the individual components, the relationship between Rab35 and MTMRs proposed in this study (and predicted by Gillingham et al.) is likely to reflect broader significance in cellular regulation beyond functions in myelination and peripheral neuropathies.

Comments:

- The localization studies shown in Figure 1e-1f (and S1) need to be quantified for changes in MTMR13 distribution to plasma membrane and to LAMP1+ compartments. As shown in S1e-S1f, for example, it is not obvious that Rab35CA necessarily increases MTMR13 recruitment to the plasma membrane. Also, labeling and text need to more clearly convey which panels are Rab35 and MTMR13 single gene overexpression versus co-expression backgrounds (is 1g and S1g with or without Rab35 overexpression?). Is there an effect from MTMR13 overexpression on Rab35 localization? Is colocailzation specific for Rab35CA versus Rab35DN, as predicted from interaction studies? Finally, is the MTMR13 PTP domain also sufficient (and necessary) for Rab35-dependent shifts in localization?

- While the initial findings are between Rab35 and MTMR13/MTMR5, the functional consequences on mTOC1 activity and other phenotypes are limited to Rab35 and MTMR2. It is compelling that MTMR13 mutants are already established to share with MTMR2 mutants CMT4B-related phenotypes. However, support is needed from a few key focused experiments aimed at testing the significance of MTMR13 as the proposed bridge between Rab35 and MTMR2 functions in their model. For example, does MTMR13 depletion also lead to hyperactive mTORC1 activity, and if so, is this rescued by MTMR2 overexpression? MTMR13 is not only an inactive phosphatase scaffold for active MTMRs, but it is also a Rab GEF with Rab-mediated endosomal-lysosomal functions. Is the MTMR13 DENN domain and GEF activity required for the proposed Rab35-MTMR13-MTMR2 pathway regulation of mTORC1 activity?

- The authors provide epistasis support for the pathway model through rescue of Rab35 and MTMR2 mutant mTORC1 activity with MTMR2 overexpression. Is this rescue dependent on MTMR2 catalytic activity?

- Both Rab35 and the MTMRs have been implicated with roles in endocytic recycling. It would be helpful for authors to provide some discussion/references about the possible significance (or not) that primary functions in these recycling roles could play in regulation of mTORC1 and myelination. Related to this point, PI(3,5)P2 is a compelling candidate for the MTMR2 substrate responsible for the phenotypes (as suggested from the Apilomod results). However, the authors’ conclusions (on page 10) should be stated more speculatively since at this point the epistasis data can only conclude dependence of the pathway on proper levels of PI(3,5)P2. It is formally possible, for example, that disrupted turnover of an MTMR2 PI3P substrate pool (such as involved in recycling) could lead to increase in a substrate pool for PIKFYVE and subsequently PI(3,5)P2 levels and effectors.

- The Rab35 KO cells show an increase in PI3P (Fig 5a-b, 6c), which the authors associate with lysosomes by the increase in mean intensity of PI3P at CD63+ compartments (Fig 5c, Fig S5c). Interestingly, the CD63+ pool also seems to be expanded (in images shown) in both Rab35 and MTMR2 depleted and co-depleted cells. Is there an increase in the number of CD63+ compartments? In addition, it might be worth normalizing PI3P to number CD63 compartments. It is theoretically possible that an increase in PI3P follows from expansion of CD63+ compartments (versus necessarily more PI3P accumulation per CD63 compartments).

- The authors should also consider/note broader context that disruption of the highly-related MTMR2 subfamily member, MTM1, also was previously associated with misregulation of mTORC1 activity.

Minor Comments:

page 2
Not clear what point is referred to here by use of “the latter”? MTMR2 subcellular localization?
“MTMR5 and MTMR13 are catalytically inactive proteins and associate with MTMR2 to potentiate phosphatase activity and to regulate its subcellular localization12-14; the latter, however, remains to be clearly defined.”

page 3
Results on BioID refer to “Tables S1, S2”, but there is no Table S2 provided.

Table S1
- What is indicated by proteins in bold font among the list of enriched proteins? There is no legend or explanation.
- What is criteria for “selective” - any value over 1?
- Please clarify distinction between Table S1a and S1b, and why there is no overlap between the lists?

Figure 1d and text
Text should more clearly communicate that “truncation mutants” refer to expression of individual domain-specific regions, and not deletions for the indicated domains (which could also be called truncation mutants).

Figure S3h-o.
What day cultures were assayed for changes in endosome/lysosome compartments?

Figure S5c
Clarify whether GFP-PI3P is GFP-2xFYVE?


Reviewer #3 (Remarks to the Author):

In this manuscript, Sawade et al probed the molecular mechanism of IPN/CMT mutations in neuropathies. They found a small GTPase, Rab35, forms a complex with MTMR13 and MTMR2, which decreases the level of PI3P and leads to inactivation of mTORC1 through the PI3K-Akt-mTOR axis. Loss of Rab35 causes mTORC1 hyperactivation and myelin abnormalities. Inhibiting mTORC1 activity or PI3P level rescues the loss of Rab35, which further supports the conclusion. This manuscript presents high quality data and it is well written. I have little to criticize and support publication on Nature Communications.

Minor concern:

Although this may go beyond the scope of this paper and the expertise of the authors, they should try to explain the molecular mechanism of how Rab35 controls the enzymatic activity of MTMR2 through MTMR13.

**Author rebuttal, first version:**

**Response to the reviewers**

We would like to thank all three referees for their careful reading of our manuscript, for their highly constructive comments, and the many thoughtful suggestions. We were very happy to learn that all three reviewers agree on the high general interest and timeliness of our study and its suitability for publication in *Nature Communications*. In the revised manuscript we have now addressed all of their questions and concerns as further detailed below.

**Responses to reviewer #1:**

In this paper Rab35 was identified as regulator of myelin growth in the peripheral nervous system by its association with the PI3-phosphatases MTMR13 and MTMR2. The authors how that the regulation occurs by mTOR signaling, thereby linking Rab35 to the known function of MTMR13 and MTMR2 to the AKT-mTORC2 signaling pathway. To arrive at this conclusion the authors use a proteome approach to identify proteins associated with Rab35-GTP followed by biochemical and cell biological assays to validate the effector molecules. In addition, a series of in vivo experiments were conducted to show the function of Rab35 deletion in myelin growth in the peripheral nervous system. The main advance of this paper is to provide a novel function of Rab35 in the nervous system. The observations are intriguing and for most parts the results are convincingly shown. However, there are a number of experiments required to fully complete the study.

Response: We thank reviewer 1 for these very positive remarks and for highlighting the conceptual advance and quality of our study. We have conducted all requested experiments as detailed below.

1. The paper jumps back and forth from Schwann cells to astrocytes. Since the in vivo function of Rab35 is shown for Schwann cells, the key experiments need to be conducted in these cells....

Response: We have considerably extended our analysis of the role of Rab35 and MTMR2 in primary Schwann cells as suggested by the referee. Specifically, we now show in the **new Figures S8e,f and S8g,h** that MTMR2 depletion in Schwann cells results in hyperactive mTORC1 signaling monitored by the pS6/total S6 ration and this correlates with elevated levels of PI 3-phosphates such as PI(3)P, i.e. substrates of MTMR2 phosphatase activity. Moreover, the **new Figure 6a,b** shows that lentiviral depletion of Rab35 in Schwann cell/ DRG neuron co-cultures results in increased mTORC1 activity similarly to Rab35 cKO nerves (Figure 6c,d). Finally, co-cultures from Rab35 cKO mice also display increased mTORC1 activity as assessed by pS6 levels (Figure 7a,c-new panels). These additional data essentially recapitulate all major findings made in HEK293T cells and in mouse astrocytes.

...and the astrocyte data could be moved to the supplement to avoid redundancy

As the analysis of primary astrocytes has been an important element in the path of discovery we prefer to keep the astrocyte data in the main manuscript. Moreover, we feel that showing a conserved role of Rab35 - MTMR complexes in controlling mTORC1 activity in different cell types including astrocytes adds to the weight and credibility of our study. We hope the referee will agree.

2. The peripheral nervous system myelin phenotype needs to be clarified. Figure 2 shows normal g-ratios and overgrowth profiles. However, the co-culture data in Figure 4 suggest hypomyelination. The authors should extend their study to analyze whether hypomyelination is also seen in vivo in mice. Early time points should be included to determine whether the onset is normal.

1

Response: As suggested by the reviewer, we have extended this analysis to early time points at P3 by measuring the number of myelinated and amyelinated fibers and by measuring g-ratio values at both P3 and P5. These data are included in the **new Figure S6**. Altogether, these data indicate that elevation of mTORC1 signaling *in vivo* does not interfere with the onset of myelination.

3. The impact of the paper would increase if the authors could study the physiological role of Rab35 during myelin development in peripheral nervous system. It is for example conceivable that Rab35 activity is important to control the termination of myelin formation. Is the activity of Rab35 altered when myelin growth comes to an end?

Response: We thank the reviewer for sharing these interesting thoughts. We indeed agree that it might be interesting to further analyze the possible role of Rab35 in the developmental control of myelination in the PNS. By performing western blot analysis on sciatic nerve lysates at different time points of postnatal development, we found that Rab35 levels are higher at early time points of postnatal development starting from P2 and then decline until P60 (**new Figure 3a**). This expression profile is similar to other negative regulators of myelination such as Dlg1 as reported in Noseda et al., J Neurosci 2013. Of note, protein levels of both Dlg1 and Pten, another negative regulator, are stabilized by axonal Nrg1 type III at early time points of myelination (Cotter et al., Science 2010). Finally, TSC1/2, a further negative regulator of the mTORC1 pathway and myelination, does not change in expression level during postnatal nerve development (Beirowski et al., PNAS 2017). These data suggest that higher expression levels of Rab35 at the onset of myelination might be necessary to titrate mTORC1 activation. Loss of Rab35, however, does not result in a dramatic increase of mTORC1 in vivo, likely because other regulators are also crucial at this stage to control mTORC1 activity.

Specific comments:

Figure 1: Panel d and e-g should be quantified.

Response: We have quantified these data as requested. These are now reported in the corresponding

figure (now Fig. 2d,e) legend.

Figure 4: Control groups are missing and need to be provided. In e and g the WT (NT) and WT

(rapamycin) are not shown.

Response: Done- these are now shown in the **new Figure 7d**.

Figure 5: the images corresponding to c should be shown.

Response: The images are shown in the **new Figure S7c** as requested.

Figure 6: the images shown in e are of low quality. It is not possible to see any signal.

Response: We have replaced these images and now include Phalloidin staining to improve the visualization of the Schwann cells (see **new Figures 10e and S8i**).

Supp Fig3: the images corresponding to g should be shown as an overview image.

Response: The image indeed is a representative uncropped example image that exemplifies the phenotype. We did not acquire low mag overview images but note that **quantitative data** are presented in the **main figure 5e**.

2

Supp FigS4: the rescue experiments using rapamycin are critical, but only shown in Supp figure, possible because the rescue was only partially successful. The authors should shift these experiments into the main figure and display them as shown in Figure 2e.

Response: We have re-arranged the figures according to the referee's suggestion. The rapamycin rescues formerly presented in fig. S4 are now shown as the **new Figure 8**.

Legends: the figure legends are too long and difficult to read. The authors should improve this to make the paper easier to read.

Response: We appreciate the comment and share the referee's concern. However, as *Nature Communications* requires us to report cell numbers and statistics in the legend there is only marginal leeway here for which we apologize. We have tried to do our best.

**Responses to reviewer #2:**

The authors here explore novel roles for Rab35 GTPase to regulate and connect together several previously known mediators of peripheral axonal myelination and health — specific MTMR phosphatases and mTORC1 kinase activity. Using BioID in HEK293T cells (and as previously reported for conserved proteins in a proteomic study by Gillingham et al), the authors identified Rab35 interactions with inactive phosphatases, MTMR13 and MTMR5. These interactions were defined by preferred binding to GTP-bound Rab35 and validated in HeLa cells. Conditional knockouts of Rab35 in mice revealed a phenocopy to MTMR13 and MTMR2 mutations previously shown with defective Schwann cells in models of CMT4B neuropathy disease. The authors explore and demonstrate a link between Rab35 and MTMR2 functions needed for downregulation of mTORC1 activity in cell cultures and in vivo, bridging previous individual findings on myelination roles for MTMR2 and mTORC1. Epistasis results support the pathway model by showing rescue of Rab35 and MTMR2 mutant mTORC1 activity with either wild type MTMR2 overexpression or inhibition of mTORC1 activity. Given the endosomal/lysosomal membrane and signaling roles already known for the individual components, the relationship between Rab35 and MTMRs proposed in this study (and predicted by Gillingham et al.) is likely to reflect broader significance in cellular regulation beyond functions in myelination and peripheral neuropathies.

Response: We thank reviewer 2 for these very positive remarks and for highlighting the conceptual advance and broad significance of our study.

Comments:

- The localization studies shown in Figure 1e-1f (and S1) need to be quantified for changes in MTMR13 distribution to plasma membrane and to LAMP1+ compartments. As shown in S1e-S1f, for example, it is not obvious that Rab35CA necessarily increases MTMR13 recruitment to the plasma membrane. Also, labeling and text need to more clearly convey which panels are Rab35 and MTMR13 single gene overexpression versus co-expression backgrounds (is 1g and S1g with or without Rab35 overexpression?). Is there an effect from MTMR13 overexpression on Rab35 localization? Is colocailzation specific for Rab35CA versus Rab35DN, as predicted from interaction studies? Finally, is the MTMR13 PTP domain also sufficient (and necessary) for Rab35-dependent shifts in localization?

3

Response: We have considerably extended our analysis of the effects of Rab35 and its nucleotide status on MTMR13 localization and vice versa. In the **new Figure 2d,e** we now show that MTMR13 associates with and is recruited by Rab35•GTP (Rab35 CA) with preference over Rab35•GDP. These data are now also quantified from large numbers of cells (**new Fig. 2e**). Furthermore, we show in the **new Figure 2f,g** that the PTP is indeed sufficient for association with and recruitment by Rab35•GTP to late endosomes/ lysosomes as predicted from the biochemical data (now shown in Figs. 1 and S1). We have also tried to improve the labeling of the panels. Indeed, Rab35 was overexpressed in former figs 1g & S1g (now Figs. 2d and S2c). Finally, we do not detect any effects of MTMR13 overexpression on Rab35 localization.

- While the initial findings are between Rab35 and MTMR13/MTMR5, the functional consequences on mTOC1 activity and other phenotypes are limited to Rab35 and MTMR2. It is compelling that MTMR13 mutants are already established to share with MTMR2 mutants CMT4B-related phenotypes. However, support is needed from a few key focused experiments aimed at testing the significance of MTMR13 as the proposed bridge between Rab35 and MTMR2 functions in their model. For example, does MTMR13 depletion also lead to hyperactive mTORC1 activity, and if so, is this rescued by MTMR2 overexpression? Response: We have gladly followed the referee's suggestion. In the **new Figures 4e,f and S4** we demonstrate that knockdown of either MTMR13 or the closely related MTMR5 by smartpool siRNAs or a single siRNA from this pool results in elevated mTORC1 signaling monitored by pS6K1/ total S6K levels (**new Figs. 4e+f, S4a,b**). Please note that the degree of mTORC1 hyperactivation is somewhat variable between experiments and conditions, but is highly statistically significant as quantified in **Fig 4c,d,f,h**. We also show that elevated mTORC1 signaling elicited by loss of MTMR13 or Rab35 is partially rescued by overexpression of catalytically active MTMR2 (**Fig. 4g,h**), whereas inactive MTMR2 is less efficient (**new Figs S4c+d)**. These data provide further support for our model according to which Rab35 recruits MTMR13/5-based phosphatase complexes, e.g. MTMR13/R2 that crucially regulate mTORC1 activity by hydrolysis of mTORC1-activating PI 3-phosphates.

MTMR13 is not only an inactive phosphatase scaffold for active MTMRs, but it is also a Rab GEF with Rab-mediated endosomal-lysosomal functions. Is the MTMR13 DENN domain and GEF activity required for the proposed Rab35-MTMR13-MTMR2 pathway regulation of mTORC1 activity?

Response: In response we have extended our biochemical analysis of Rab35-MTMR13 complex formation. In the **new Figure 1d,e** we show that mutant MTMR13 lacking its DENN domain co-immunoprecipitates with active Rab35•GTP with similar specificity and efficiency than the full-length MTMR13 protein. Moreover, MTMR13 or the PTP domain of MTMR13 alone are efficiently recruited to late endosomes or lysosomes by active Rab35•GTP but not by inactive Rab35•GDP as shown in the **new Figures 2d-g**. These results further confirm that MTMR13 acts as a Rab35 effector rather than a Rab35 GEF. That said, these data do not exclude the possibility that the DENN domain of MTMR13 could serve as a GEF for Rabs other than Rab35. Future studies will need to address this possibility.

We have also tried to address the role of MTMR13's DENN domain in mTORC1 regulation based on MTMR13 knockdown (that confirm its role in mTORC1 signaling as alluded to above; see **new Figures 4e,f and S4**) experiments. Due to the large size of the MTMR13 cDNA (about 5.5 kb) transfection efficiencies with any method that we have tried are too low to achieve functional rescue while the cDNA is also too large to be packaged into lentiviral vectors used in our lab. These caveats have precluded further functional studies of MTMR13. We hope that given the wealth of new data provided the referee

4

will agree that our study nonetheless may open the door for more detailed analyses to be conducted in the future.

- The authors provide epistasis support for the pathway model through rescue of Rab35 and MTMR2 mutant mTORC1 activity with MTMR2 overexpression. Is this rescue dependent on MTMR2 catalytic activity?

Response: We show in **Figs. 4g,h** and the **new S4c,d** that elevated mTORC1 signaling elicited by loss of MTMR13 or Rab35 is partially rescued by overexpression of catalytically active MTMR2 (**Fig. 4g,h**), whereas inactive MTMR2 appears to be less efficient (**new Figs S4c+d)**.

- Both Rab35 and the MTMRs have been implicated with roles in endocytic recycling. It would be helpful for authors to provide some discussion/references about the possible significance (or not) that primary functions in these recycling roles could play in regulation of mTORC1 and myelination. Related to this point, PI(3,5)P2 is a compelling candidate for the MTMR2 substrate responsible for the phenotypes (as suggested from the Apilomod results). However, the authors’ conclusions (on page 10) should be stated more speculatively since at this point the epistasis data can only conclude dependence of the pathway on proper levels of PI(3,5)P2. It is formally possible, for example, that disrupted turnover of an MTMR2 PI3P substrate pool (such as involved in recycling) could lead to increase in a substrate pool for PIKFYVE and subsequently PI(3,5)P2 levels and effectors.

Response: We agree and acknowledge the possibility that Rab35-MTMRs may act, at least in part, via effects on endocytic recycling in the results (p. 9, including a potential role for PI(3)P) and discussion sections (p.10/11) of our revised Ms. The revised discussion on p.10/ 11 with respect to the role of Rab35 in the control of mTORC1 activity and, thereby, myelination now reads as follows:

*"How exactly the different forms of overgrown myelin such as myelin outfoldings and tomacula arise, remains poorly understood. Akin to results for PTEN mutants^22^ we found that mTORC1 inhibition ameliorated tomacula but much less well myelin outfoldings in Rab35 cKO^SC^ mice, suggesting that mTORC1 activity primarily controls bulk membrane addition. It is therefore conceivable that other pathways beyond mTORC1 signaling contribute to focal hypermyelination in Rab35 cKO^SC^ mice. These may include dysregulation of exosome secretion, a process known to be important for myelin membrane growth that is regulated by the Rab35 GTPase activating proteins TBC1D10A-C^34^, phosphoinositide regulation of actin dynamics by Rab3529, 35, 44, 80 and/ or MTMRs, or the endocytic recycling of signaling receptors, for example as a consequence of alterations in the activity of the small GTPase Arf6 29, 35, 44, 80 via the Rab35 effector ACAP228, 29, 41, 45. "*

- The Rab35 KO cells show an increase in PI3P (Fig 5a-b, 6c), which the authors associate with lysosomes by the increase in mean intensity of PI3P at CD63+ compartments (Fig 5c, Fig S5c). Interestingly, the CD63+ pool also seems to be expanded (in images shown) in both Rab35 and MTMR2 depleted and co-depleted cells. Is there an increase in the number of CD63+ compartments? In addition, it might be worth normalizing PI3P to number CD63 compartments. It is theoretically possible that an increase in PI3P follows from expansion of CD63+ compartments (versus necessarily more PI3P accumulation per CD63 compartments).

Response: The referee indeed is right. We observe both elevation of PI(3)P content and an expansion of the endolysosomal compartment marked by CD63. The levels of PI(3)P reported in **Fig. 9c** (formerly Fig

5

5c) have been normalized to the CD63-positive area and, thus, take the observed expansion of CD63-positive organelles into account. Hence, PI(3)P levels are increased within late endosomes/ lysosomes.

- The authors should also consider/note broader context that disruption of the highly-related MTMR2 subfamily member, MTM1, also was previously associated with misregulation of mTORC1 activity. Response: We have modified our discussion on p. 11 to allow for the possibility that other MTMRs may also regulate mTORC1 signaling. In this context we note that conflicting data with respect to the phenotype of MTM1 loss on mTORC1 signaling have been reported in different models. The revised text reads as follows:

*"Consistent with this hypothesis we observe that focal hypermyelination in peripheral nerves from Rab35 cKO^SC^ mice is even more severe than in Mtmr2 or Mtmr13 KO models59, 60, 61, 62. Rab35-bound MTMRs (e.g. including MTMR13, MTMR5 and their associated active MTMs such as MTMR2, MTMR1, and possibly others) in this model would counteract the role of the PI(3,5)P2-metabolizing PIKFYVE-Vac14-FIG4 complex, a genetic interactor of Mtmr2^11^. Indeed, PIKFYVE-Vac14-FIG4 are required for oligodendrocyte differentiation and myelination in the central nervous system11, 82, 83. "*

Minor Comments:

page 2

Not clear what point is referred to here by use of “the latter”? MTMR2 subcellular localization?

“MTMR5 and MTMR13 are catalytically inactive proteins and associate with MTMR2 to potentiate

phosphatase activity and to regulate its subcellular localization12-14; the latter, however, remains to be

clearly defined.”

Response: Thank you! This has been re-phrased in the revised Ms text.

page 3

Results on BioID refer to “Tables S1, S2”, but there is no Table S2 provided.

Response: We apologize for the mistake. In fact, there is a single Excel table S1 with two slides that

should have been referred to as Tables S1a and S1b. This has now been corrected in the revised Ms text.

Table S1

- What is indicated by proteins in bold font among the list of enriched proteins? There is no legend or

explanation.

Response: These are now explained in the legend.

- What is criteria for “selective” - any value over 1?

- Please clarify distinction between Table S1a and S1b, and why there is no overlap between the lists? Response: This has been clarified in the legend and is now also explained in the methods section. Selectively enriched proteins (reported in Table S1a) were ONLY detected in the BirA-Rab35 sample but not in the negative control. Table S1b shows proteins enriched more than five-fold in the BirA-Rab35 sample over the negative control.

Figure 1d and text

Text should more clearly communicate that “truncation mutants” refer to expression of individual

6

domain-specific regions, and not deletions for the indicated domains (which could also be called

truncation mutants).

Response: Thank you- done.

Figure S3h-o.

What day cultures were assayed for changes in endosome/lysosome compartments?

Response: This is now described clearly in the methods (i.e. DIV 20-22).

Figure S5c

Clarify whether GFP-PI3P is GFP-2xFYVE?

Response: This has been corrected in the revised suppl. figure.

**Responses to reviewer #3:**

In this manuscript, Sawade et al probed the molecular mechanism of IPN/CMT mutations in neuropathies. They found a small GTPase, Rab35, forms a complex with MTMR13 and MTMR2, which decreases the level of PI3P and leads to inactivation of mTORC1 through the PI3K-Akt-mTOR axis. Loss of Rab35 causes mTORC1 hyperactivation and myelin abnormalities. Inhibiting mTORC1 activity or PI3P level rescues the loss of Rab35, which further supports the conclusion. This manuscript presents high quality data and it is well written. I have little to criticize and support publication on Nature Communications.

Response: We thank the reviewer for these highly positive remarks and for his/ her strong support to publish our work in Nature Communications.

Minor concern:

Although this may go beyond the scope of this paper and the expertise of the authors, they should try to explain the molecular mechanism of how Rab35 controls the enzymatic activity of MTMR2 through MTMR13.

Response: Our data support a model - further strengthened by additional experiments shown in the new Figure 2 - according to which Rab35•GTP recruits heterodimers of MTMR13 (and likely the related MTMR5) with active MTMRs, i.e. MTMR2, to late endosomal/ lysosomal compartments to facilitate hydrolysis of PI(3)P and PI(3,5)P2. As full-length recombinant MTMR13 and MTMR2 are currently unavailable we unfortunately cannot address whether Rab35 in addition might regulate MTMR activity. As Rab35 does not directly associate with the active MTMR subunit, i.e. MTMR2, we consider this unlikely, although certainly not impossible.

**Reviewer comments, second version:**

REVIEWERS' COMMENTS:

Reviewer #1 (Remarks to the Author):

The authors have done a perfect job in addressing all major issues raised by the reviewers. I recommend publicatioin


Reviewer #2 (Remarks to the Author):

The authors have satisfactorily addressed the necessary data analysis and reviewers' questions. The manuscript has been improved by new results that demonstrate MTMR13 PTP domain function and MTMR2 epistasis in the proposed pathway for mTORC1 regulation, as well as the addition of more data in Schwann cells.

The authors should be cautious in their interpretation of MTMR2 catalytic activity involvement based on data shown in Figure S4d, since any differences in mTORC1 activity between conditions is not striking in this panel. A minor point is that there’s a typo on Figure 6b.

**Author rebuttal, second version:**

**Response to the reviewers**

We would like to thank all three referees for carefully reviewing our revised manuscript and for their support to publish our findings in *Nature Communications*. We have now addressed their remaining questions as detailed below.

**Responses to reviewer #1:**

The authors have done a perfect job in addressing all major issues raised by the reviewers. I recommend publication.

Response:

We are glad to hear that the reviewer approves of our revised manuscript and thank the reviewer for his/ her support to publish our findings in *Nature Communications.* No further action was necessary.

**Responses to reviewer #2:**

The authors have satisfactorily addressed the necessary data analysis and reviewers' questions. The manuscript has been improved by new results that demonstrate MTMR13 PTP domain function and MTMR2 epistasis in the proposed pathway for mTORC1 regulation, as well as the addition of more data in Schwann cells.

Response:

We thank the reviewer for his/ her positive remarks and the support to publish our findings in *Nature Communications.*

The authors should be cautious in their interpretation of MTMR2 catalytic activity involvement based on data shown in Figure S4d, since any differences in mTORC1 activity between conditions is not striking in this panel. A minor point is that there’s a typo on Figure 6b.

Response:

In agreement with the referee's comment we amended our reference to Figure S4d, which now reads as follows:

*"In contrast, inactive MTMR2 was* ***less effective*** *at rescuing elevated mTORC1 activity in cells depleted of MTMR13 (Supplementary Figure 4c) or Rab35 (Supplementary Figure 4d)."*

We note that in addition to the experiment shown in Suppl. Fig. 4d we provide multiple additional independent lines of evidence in support of a key role for MTMR2 activity in the regulation of mTORC1 signaling and focal hypermyelination, e.g. rescue of both mTORC1 hyperactivation and increased myelin biogenesis in the presence of Vps34 or PIKFYVE inhibitors, see e.g. Figures 9 and 10.

We have also corrected the typo in Figure 6b - thank you!

**Reviewer comments, third version:**

**Author rebuttal, third version:**
